# Gender and equity within antimicrobial resistance: a scoping review of evidence and policy in Bangladesh and Sri Lanka

**DOI:** 10.1186/s12889-026-27842-y

**Published:** 2026-06-04

**Authors:** Sneha Paul, Farha Musharrat Noor, Syeda Tahmina Ahmed, Nahitun Naher, Bachera Aktar, Naushalya Rajapaksha, Thiraj Haputhanthri, Sally Theobald, Rosie Steege, Katy Davis, Meenakshi Monga, Sabina Faiz Rashid, Syed Masud Ahmed

**Affiliations:** 1 BRAC James P Grant School of Public Health, BRAC University, Dhaka, Bangladesh; 2 World Health Organization Sri Lanka, Colombo, Sri Lanka; 3 Liverpool School of Tropical Medicine, Liverpool, UK

**Keywords:** Antimicrobial resistance, Gender, Equity, Policy, Bangladesh, Sri Lanka, Scoping review

## Abstract

**Background:**

Antimicrobial resistance (AMR) is a significant public health concern, disproportionately affecting Low-and-Middle-Income countries (LMICs) across Southeast Asia, including Bangladesh and Sri Lanka. This WHO region faces a high burden of AMR, which is driven by social, biological, and structural factors. Social determinants such as poverty, education, healthcare access, and gender roles and norms contributed significantly to the non-recommended use of antimicrobials. Despite this growing burden, there is limited research on the social dimensions of AMR, including how gender norms and disparities shaped AMR in Bangladesh and Sri Lanka.

**Purpose:**

This scoping review investigated how gender and equity interact with intersectional factors, including biology, age, and socioeconomic status, as well as complex power structures shaping AMR in Bangladesh and Sri Lanka, and assessed gender and equity inclusion within AMR national policy, guidelines, reports, and the National Action Plan.

**Methods:**

PubMed, Scopus, Google Scholar, and Index Medicus for South-East Asia Region (IMSEAR) were searched for peer-reviewed articles, and institutional websites were searched for grey literature published in English between 2014 and 2024. Data was extracted, charted, and analyzed through a thematic framework by using an intersectional lens and WHO Gender-responsive Assessment Scale. Our review followed the Preferred Reporting Items for Systematic Reviews and Meta-Analyses protocol (PRISMA-P) and its Extension for Scoping Reviews (PRISMA- ScR) checklist.

**Results:**

This review identified 60 studies (38 from Bangladesh, 22 from Sri Lanka) and 30 grey documents; including policy, National Action Plans (NAPs), guidelines, and reports. Of the empirical studies, 53 (88%) used quantitative methods, 5 were qualitative, and 2 used mixed methods, with 47 studies (78%) focused on human health. Among the included studies, 70% (42) reported gender-related differences in AMR knowledge, attitudes, practices, exposure, or access, and 45% (27 studies) highlighted reliance on informal drug sellers.

Using an intersectional lens, this study revealed a significant impact of the complex interplay between gender, social determinants, and structural inequalities. Women's AMR exposure and barriers to recommended antimicrobial use were shaped by their high biological susceptibility, limited healthcare access, financial dependency, limited decision-making autonomy, caregiving roles, and reliance on informal providers. Men’s AMR risk and barriers to recommended antimicrobial use were influenced by their occupational exposure, over-reliance on informal providers, past experience, social-network influence, and easy access with financial and decision-making autonomy. Additionally, this review identified critical gaps, including a lack of gender-disaggregated data and the absence of gender and equity dimensions in national documents and policies.

**Conclusion:**

The findings highlighted the importance of incorporating gender-responsive indicators in surveillance systems, and targeted context-specific awareness campaigns must be integrated into AMR research, policy, and healthcare systems to identify inequities and effective implementation.

**Supplementary Information:**

The online version contains supplementary material available at 10.1186/s12889-026-27842-y.

## Introduction

Antimicrobial agents include antibiotics, antivirals, antifungals, and antiparasitics, essential for preventing and curing infectious diseases in humans, aquatic and terrestrial animals, and plants [1]. Antimicrobial resistance (AMR) develops when bacteria, viruses, fungi, and parasites diversify over time, and stop responding to medications, making diseases more difficult to cure. The growing burden of antimicrobial resistance is exacerbated by antibiotic use in human and animal populations, which in turn are consequences of complex social factors like poverty, lower education level, unemployment, and limited healthcare access [2]. The increasing prevalence of AMR has reduced the effectiveness of many first line antibiotics, which has led to catastrophic treatment costs, limited overall access of required antibiotics, and ultimately resulted in treatment failure and higher mortality from bacterial infections [1].

The World Health Organization (WHO) recently declared AMR as one of the 10 global public health threats [1]. Between 2025 and 2050, AMR is estimated to have caused 39.1 million deaths directly and 169 million deaths indirectly [3]. It is predicted that, by 2050, there will likely be 46.5 million years of healthy life lost, directly attributable to AMR. This is up from 42.6 million in 2022 [3]. Furthermore, a recent study projects that if AMR accelerates, global Gross Domestic Product (GDP) could fall by up to USD 1.67 trillion by 2050 [4], and low- and middle-income countries (LMICs) will bear a disproportionate share of the economic impact [5].

According to projections, South Asia, Southeast Asia, East Asia and Oceania, and sub-Saharan Africa would bear the highest burden of AMR. AMR is expected to cause a total of 11.8 million deaths in South Asia, 8.96 million in Southeast Asia, and 6.63 million in sub-Saharan Africa between 2025 and 2050 [3]. By 2050, AMR might cost Asia between USD 550 and USD 700 billion of the region’s GDP. It may also significantly impact health systems, increasing treatment expenses by between 2.5% and 10% of overall healthcare expenditure [5]. Among 204 countries, Bangladesh and Sri Lanka rank 75th and 58^th,^ respectively, in terms of death rates associated with antibiotic resistance [6]. In 2019, Bangladesh and Sri Lanka faced 26,000 and 2,300 deaths respectively directly from AMR and 98,800 and 8,800 deaths were associated with AMR [6].

AMR in Bangladesh and Sri Lanka is exacerbated by factors such as barriers to the recommended use of antibiotics in human, agriculture, and veterinary sectors, including lack of access to clean water, sanitation, and hygiene (WASH) [1]. Antimicrobials are widely available in these contexts as over the counter (OTC) medicines at local medicine stores and community pharmacies [7, 8] and the population face barriers to access to healthcare services - compounded by a lack of affordable, high-quality healthcare facilities. Social practices and structural barriers are often driving and shaping AMR.

Gender, a key social determinant of health, refers to “the roles, relationships, and norms that are socially constructed and assigned to men and women, boys and girls, transgender individuals, and non-binary individuals by a culture” [9]. In contrast, sex refers to the biological or chromosomal attributes assigned at birth and may differ from a person’s gender identity, but sex identifies and distinguishes individuals as males, females, and intersex people [10]. AMR intersects in multiple complex and context-specific ways with sex, gender, and equity [11]. Susceptibility to resistant infections is influenced by biological sex, including anatomical factors, such as during pregnancy, childbirth, abortion, and the post-abortion period [11]. Gender also intersects with biology in driving AMR as women’s body anatomy, poor menstrual hygiene, sexual abuse, and other trauma, they are more likely to experience urinary tract infections (UTIs) [12] and consume more antibiotics during their lifetime than men do, especially during pregnancy, child birth caesarean births, to decrease menstrual cramps [13, 14]. Additionally, women are more likely to come into contact with infections during caregiving to ill family members, taking care of domestic animals, maintaining gardens, and other household tasks, and women’s access to healthcare is impacted by deeply rooted gendered social norms and disparities that limit women’s mobility, financial autonomy, and decision-making power [11, 15]. Gender-disaggregated data from different regions show gender differences in AMR across different contexts, but data availability in Southeast Asia is uneven. For example, in Europe, due to UTIs and reproductive health, women consume more antibiotics than men, while in Africa, men show higher rates of drug-resistant TB [16]. Identifying these interconnected social factors is essential for developing effective and equitable interventions that target the root causes of AMR and promote global health security. To date, exploring how the social determinants influence AMR has been limited.

We conducted a scoping review on gender and equity to understand how gender norms and disparities, and broader power structures in Bangladesh and Sri Lanka contribute to the growing AMR crisis. Bangladesh and Sri Lanka were chosen purposively based on their epidemiological significance, policy value, and comparative worth in the South Asian context. The rate of antimicrobial resistance (AMR) in both countries is significant and one of the highest AMR burdens in the world [3, 6]. Meanwhile, they provide valuable differences in healthcare systems, access to healthcare, and socio-cultural and gender norms that influence antimicrobial use practices [8, 17]. The National Action Plans of both countries are also consistent with the WHO Global Action Plan on AMR, which gives both countries a policy context to evaluate the consideration of gender and equity [18–22], even though it is shown that gender and equity are not well-considered in AMR research and policy [11, 16]. Moreover, they were chosen according to the priorities of the Gender and Equity within Antimicrobial Resistance (GEAR-up) consortium that allows conducting a comparative analysis policy-relevant and contextually-grounded.

Our review will help develop more inclusive, adaptable, and context-specific approaches to combating AMR by revealing underlying disparities.

## Methods

**Fig. 1:**
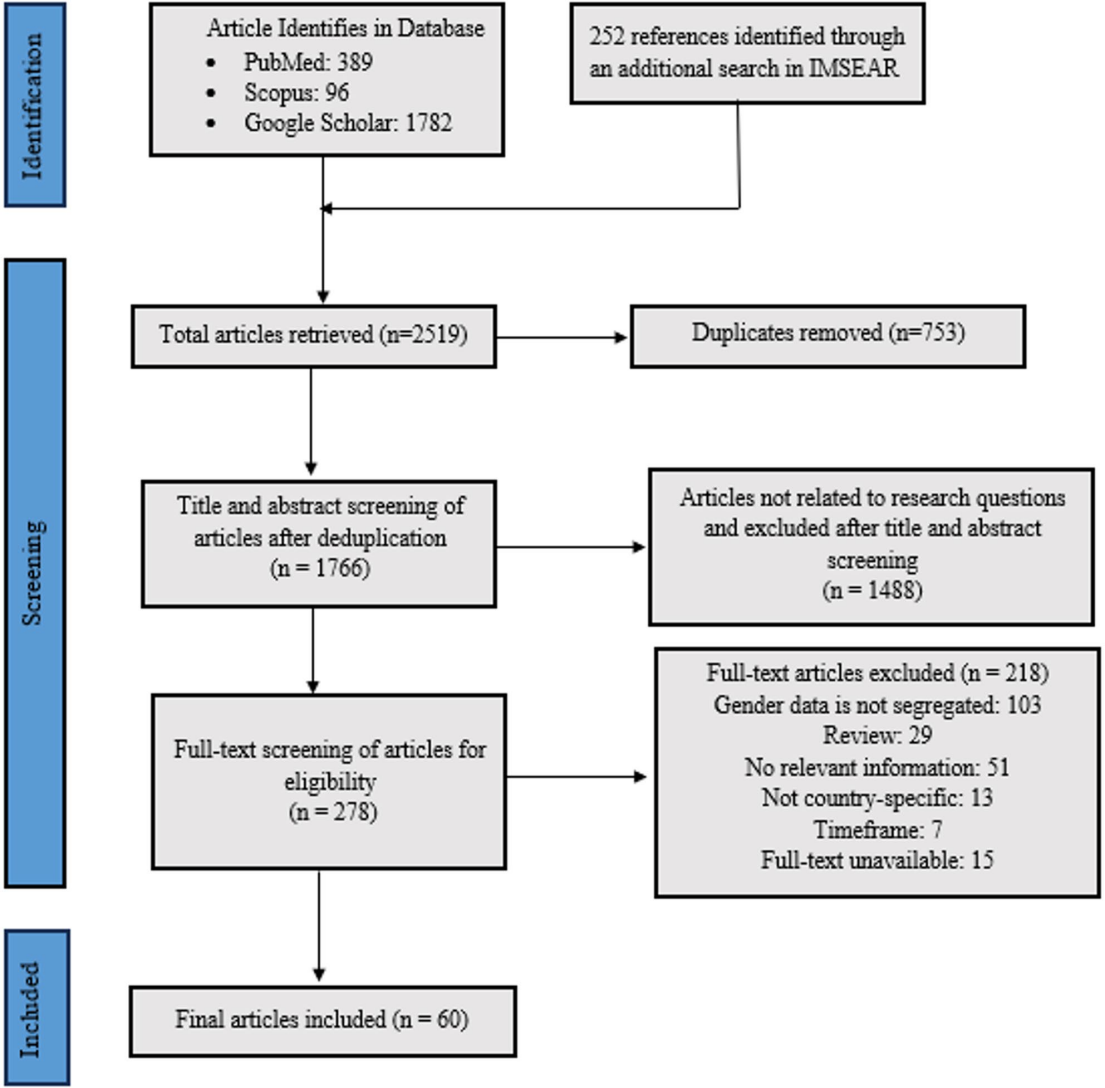
PRISMA flow diagram

We conducted this scoping review following the Preferred Reporting Items for Systematic Reviews and Meta-Analyses Protocol (PRISMA-P) and its Extension for Scoping Reviews (PRISMA- ScR) checklist [23]. This review was a part of work under the Gender and Equity within Antimicrobial Resistance (GEAR up) consortium, funded by the Department of Health and Social Care (DHSC)’s Fleming Fund. Bangladesh and Sri Lanka were priority countries for GEAR up, so this review included articles and grey documents, including policy, NAP (National Action Plans), guidelines, and reports from Bangladesh and Sri Lanka. For selecting articles on gender and equity, relevant documents like peer-reviewed articles, NAPs, strategies, policies, frameworks, guidelines, and practices within AMR were screened and reviewed (Fig. 1). Grey literature was identified through a systematic search of institutional and governmental websites, such as Ministries of Health, national regulatory bodies and global bodies like WHO and partners that undertake AMR programs. Key AMR-related search terms were used in a predefined source list of Bangladesh and Sri Lanka. Relevant national documents publicly available were incorporated and reference lists were screened. Studies were chosen independently by two reviewers who resolved any discrepancies by discussing them. We applied an intersectionality lens to explore how many axes of identity, such as gender, occupation, and socioeconomic status, intersect to produce different kinds of privilege or marginalization in terms of health outcomes [24]. We used intersectionality to explore the evidence of how gender interacts with several individual factors, including biology, age, and socioeconomic status, as well as complex power structures, to influence the determinants of AMR. This approach allows for a greater understanding of the ways AMR impacts various population groups in various geographic settings. In order to identify antimicrobial-related vulnerability, healthcare access, and non-recommended antimicrobial use across different population groups, we explored how gender interacts with other factors like occupation, socioeconomic status, biological factors, and age during thematic analysis and data extraction. In addition to this, we explored the level of gender and equity integration of each document by using the “WHO Gender-responsive Assessment Scale” (Additional Files 4, & 6) [25]. This scale categorized documents into five levels, including gender unequal, gender-blind, gender-sensitive, gender-specific, and gender-transformative, where level 1 means reinforces gender inequality in policies and level 5 means it addresses the root causes of gender-based inequalities by focusing on strategies that properly address gender and equity dimensions, and the detailed scoring criteria for the Gender-responsive Assessment Scale is given in Additional File 8. Each document was independently reviewed and scored by two researchers using the WHO Gender-responsive Assessment Scale (Additional Files 4, 5, 6, 8), which categorizes policies from gender-blind to gender-transformative, as discussed above and Additional File 8. The inter-rater agreement was evaluated by comparing the independent scores and indicated a high level of consistency between reviewers; disagreements were resolved through discussion and consensus between two reviewers, with consultation from a third reviewer when necessary.

### Search strategy and selection criteria

We developed a protocol (Table 1) that specified the review questions, inclusion/exclusion criteria, data sources, and time frame to be used. This search strategy followed the PCC framework (Population, Concept, and Context). Here, for each category (Population/concept/Content), we used OR to combine synonyms and related terms. Then, we used AND to connect different categories. For example, Population AND Concept 1 AND Concept 2 AND Concept 3 AND Context (Context 1/Context 2) (Table 2). We finalized keywords for AMR and gender disparities after discussion among the team members, and a pilot search was done in PubMed and Scopus. All the search strategies presented in Table 2 and key search terms for PubMed, Scopus, Google Scholar, and IMSEAR are presented in Additional File 1. For each search conducted in Google Scholar, results were sorted by relevance, and we screened studies from the first 10 pages of results. This approach was used because Google Scholar provides a very large number of results, which is commonly used in scoping and systematic reviews.

**Table 1:** Literature review protocol

Objectives	● To investigate how gender, equity, and intersectional factors such as occupation, financial condition, educational status, age, anatomical structure influence health seeking behaviour, as well as knowledge, attitudes, and practices (KAP) related to antimicrobial resistance (AMR) in Bangladesh and Sri Lanka. ● To explore whether the policy, NAP law, guideline, and report, frameworks in Bangladesh and Sri Lanka addressed AMR and focused on gender and equity considerations and identified the research gaps.	
Review Questions	● What evidence is available and what knowledge gaps exist regarding the influence of gender roles, norms, and equity disparities on knowledge, attitude, and perception regarding AMR? ● What intersectional factors (socioeconomic status, location, education, occupation) interplay with gender disparities to influence AMR issues in Bangladesh and Sri Lanka? ● What evidence and knowledge gaps exist regarding the role of healthcare providers (HCPs) (formal and informal) from a gendered perspective in tackling rising AMR in Bangladesh and Sri Lanka? ● What power dynamics and gender norms influence decision-making processes related to healthcare-seeking behavior and medication uptake in the context of AMR? ● What legal and policy frameworks, including a national action plan, are available regarding gender and equity within AMR in Bangladesh and Sri Lanka?	
Search Strategy	Inclusion criteria	AMR and gender-related studies conducted in Bangladesh and Sri Lanka (AMR-related knowledge and practice, decision-making power, health-seeking behavior, existing policies and strategies, health security studies that acknowledge and address AMR as a global threat)
		Full-text articles, peer-reviewed articles, grey materials (report, policy, NAPs, etc. from institutional website)
		Language: English
		Timeframe: 2014–2024
	Exclusion Criteria	Excluded studies other than those from Bangladesh and Sri Lanka, and studies that do not focus on AMR and gender equity.
Data sources	● Peer-reviewed articles: PubMed, Scopus, Google Scholar (included first 10 pages), and IMSEAR ● Grey materials: Institutional websites of Bangladesh and Sri Lanka	

**Table 2:** Search strategy

SL	PCC	Search Term
1	(a) Population (combined by ‘OR’)	Vulnerable groups, Women, Adolescent, Children, Elderly, Youth, Poor people, Homeless people, Slum Dwellers, slum settlements, Informal settlements, Marginalized people, Minorities, Unemployed, Low-income people, Daily wage laborers, Female-headed households, widows, Displaced people, migrant people, climate-induced displacement, Climate affected migrants, Frontline health workers, sex worker, Community pharmacist, Farmer, Animal husbandry, Transgender, women lead household, pregnant women, Caregivers
2.	(b) Concept 1 (AMR) (combined by ‘OR’)	Antimicrobial resistance, Antibiotic resistance, Drug resistance, Microbial resistance, AMR, Drug Uptake, Medicine uptake, purchasing medicine, Self-medication
3.	(c) Concept 2 (Gender & Equity) (combined by ‘OR’)	Gender, Equity, Male, Female, Man, Women, sex, Gender disparity, Social inequity, Social disparity
4.	(d) Concept 3 (content) (combined by ‘OR’)	Study approach, Methodology, Framework analysis, survey questionnaire, guidelines, Policies, SoPs, Operational plan, National action plan, intervention, implementation, health-seeking behavior, decision-making, Access to health-care facilities, prescription pattern, Antimicrobial-use, messaging, policies, intervention, health practices
5.	(e) Context 1	Bangladesh
	a, b,c, d,e groups were combined with Boolean operator ‘AND’	
6.	(f) Context 2	Sri Lanka
	a, b,c, d,f groups were combined with Boolean operator ‘AND’	

We included articles assessing the relation between AMR and gender and other intersectional factors, such as occupation, financial condition, educational status, age, and anatomical structure. We included the human, animal, and environment sectors, which are closely related to human health, addressing AMR. All types of peer-reviewed research papers were included; however, review papers, protocols, conference abstracts, commentaries, and newsletters were excluded. Grey materials, such as NAPs, policies, reports, and guidelines related to AMR were included. Articles focusing on other countries rather than Bangladesh and Sri Lanka were excluded. We included peer-reviewed articles and grey literature published between 1st January 2014 to 31 December 2024 written in English. This timeframe was purposively selected to capture significant developments in AMR governance and the health system in Bangladesh and Sri Lanka. The starting points align with major infectious disease outbreak, such as Nipah and Zika, which significantly influence one health surveillance system, subsequently shaping National AMR Governance. Additionally, this timeframe aligns with major global initiatives, for example, the WHO Global Action Plan on AMR 2015 [18]. The endpoint (December 2024) was selected to reflect the most recent complete body of evidence before ongoing policy developments. This review was conducted as a part of the larger gender mainstreaming study under the GEAR up consortium, which informed the following qualitative studies and gender and equity inclusion in the national AMR policy documents in 2025, including the draft National Antimicrobial Resistance Surveillance Strategy of Bangladesh (2025–2030). The scope of this review was limited to academic and grey literature published up to the end of 2024 to ensure a clear analytical boundary between evidence synthesis and its application to policy development.

We concentrated on English-language peer-reviewed articles and policy documents, as our multi-country paper has co-authors from Bangladesh, Sri Lanka, and the UK. Using English ensured consistency across our research team, which shared a common understanding. Additionally, most national-level policy documents in Bangladesh and Sri Lanka are available in English. To ensure the conceptual relevance, the search strategy required that the main domains (population, AMR, gender and equity, and context) be present simultaneously. While this structured approach may have narrowed the range of retrieved studies. However, the risk of excluding the studies was minimized through several measures. We used multiple databases, extensive synonym lists, manual search, and screening of the titles, abstracts, and reference lists of the articles to reduce the risk of excluding relevant studies.

### Article selection process

All the records identified from the database search were exported to EndNote (version 21.5) for removing duplicates and finally uploaded to Rayyan from EndNote for screening [26]. The remaining articles were screened in two stages. In the first stage, the title and abstract were screened in Rayyan based on some predetermined inclusion criteria by two independent reviewers. After that, potential articles were moved forward for full-text screening and screening done in Rayyan. In the second stage, the same two reviewers independently screened the full texts of the selected articles to determine their eligibility for final inclusion in the analysis. Reasons for exclusion were documented at both screening stages. Any discrepancy regarding the inclusion of an article was resolved by cross-checking and discussion between the two reviewers, and a third reviewer was consulted when required. The number of articles at each stage, including articles identified, duplicates removed, titles and abstracts screened, full texts assessed, and final inclusion of the articles, was recorded, and the process was summarized in a PRISMA-ScR flow diagram to ensure the transparency and reproducibility of the selection process.

### Data extraction and analysis

Thematic analysis was done in a inductive-deductive approach. The preliminary coding framework was created in accordance to the review questions and literature on gender and AMR. In data extraction, the codes were successively narrowed down to include new patterns in the data and created sub-themes under every boarder themes by team discussion. We created a data extraction table in Excel and extracted data manually by two independent reviewers. Data was extracted for the variables including author name, year of publication, country, study type, study design/methodology, Journal or website information, and main findings under the key themes given in data extraction form Additional file 9 [11]. Any differing opinions on finalizing thematic and sub-thematic categorization were resolved by the discussion among the team members under the supervision of the principal investigator. The data extraction and analysis were driven by an intersectionality-informed approach that recognizes the interplay of multiple social factors, including gender, socioeconomic status, geographic location, and occupation, to determine health outcomes and policy experiences in AMR. During data extraction, we systematically collect information related to these intersecting factors rather than treating them as independent variables. In the analysis phase, the development of themes was based on the examination of how these social categories were overlapping and impacted vulnerabilities, access to resources, and representation in the AMR policy processes. Also, a “mixed studies review” approach was used for data extraction [27].

Consistent with scoping review methodology, formal quality appraisal was not conducted.

## Results

For this scoping review, we used Google, PubMed, Scopus, Google Scholar, and IMSEAR databases to search relevant literature from Bangladesh and Sri Lanka. A total of 2519 articles were identified. After eliminating duplicates, 1766 articles were screened for title and abstract screening with the inclusion criteria. In the screening, 278 articles yielded the criteria for full-text screening. We finally included and analyzed 60 articles in this review (Fig. 1: PRISMA Flow Diagram). We reviewed a total of 30 documents, including NAPs, strategic frameworks, guidelines, and reports for both Bangladesh and Sri Lanka (Additional Files 4, 5 & 6).

## Characteristics of the included studies

**Fig. 2:**
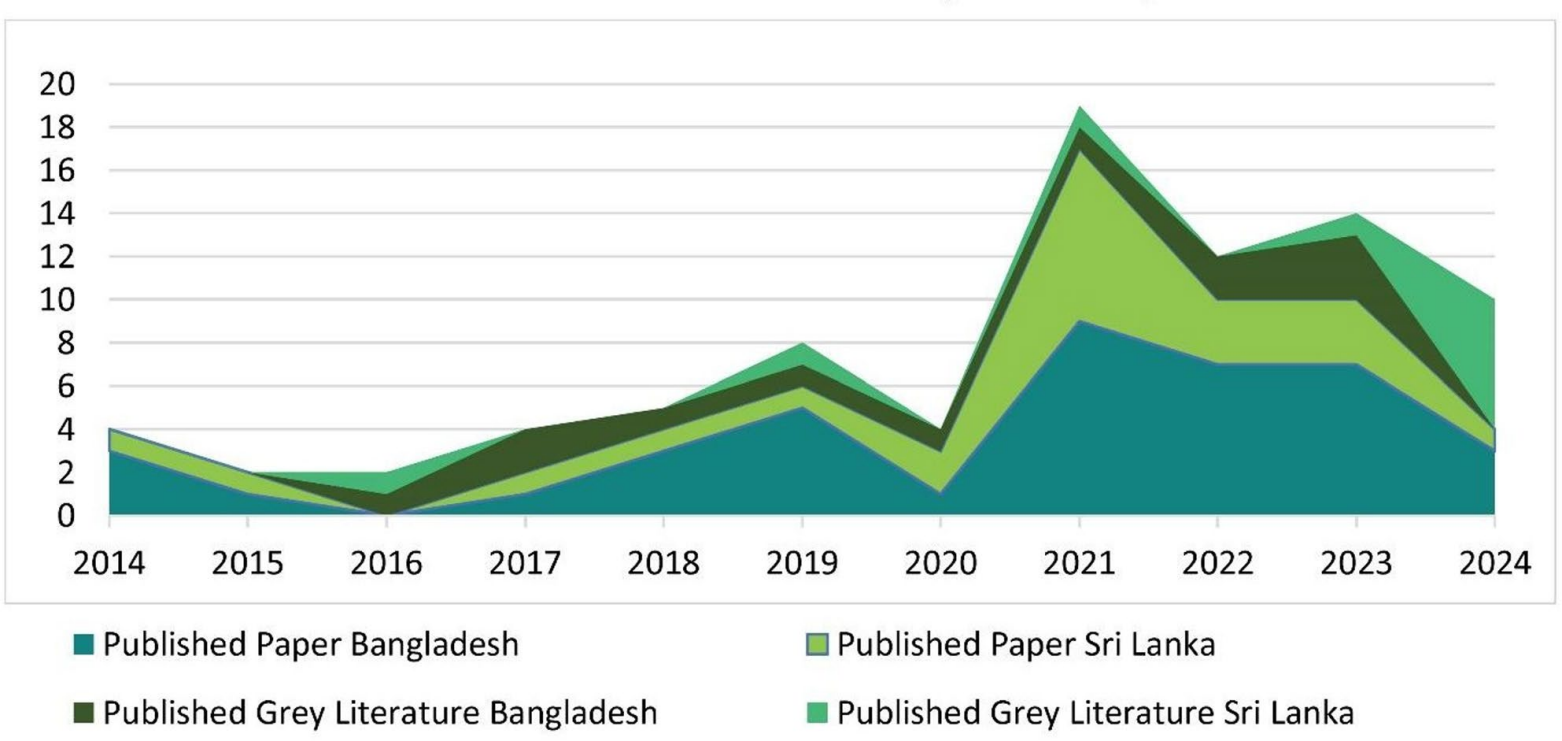
Annual publication trends (2014–2024)

**Fig. 3:**
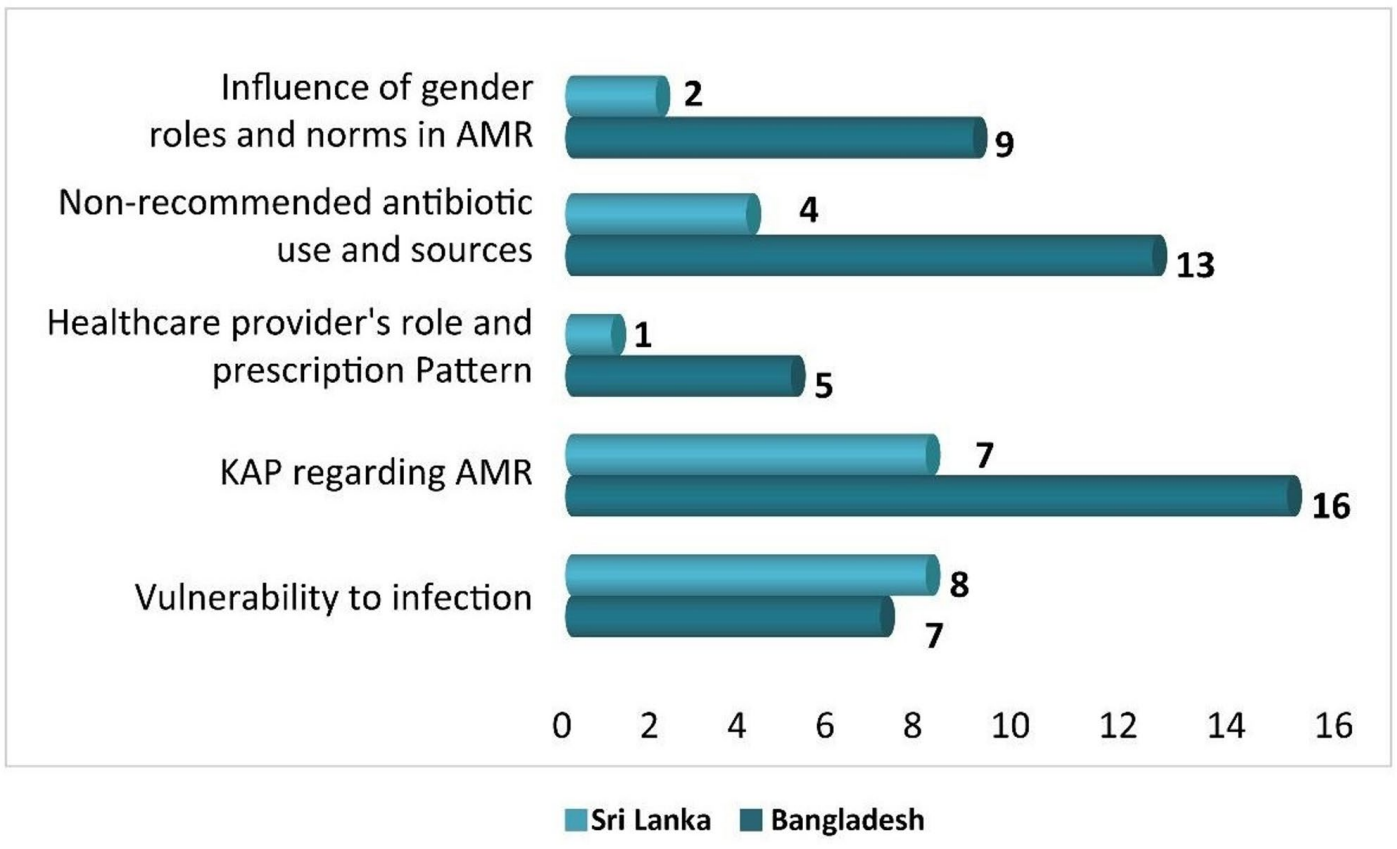
Distribution of studies by country and themes

The majority of the articles came from Bangladesh (38 from Bangladesh; 22 from Sri Lanka). Among them, 47 studies focused on human health, and 13 addressed animal and environmental health. Additionally, 5 qualitative, 2 mixed-method studies, and 53 quantitative studies were included (Additional File 2 & 3). Bangladesh and Sri Lanka both had the highest number of peer-reviewed articles and grey literature in 2021 (Fig. 2). Most studies focused on non-recommended antimicrobial use and knowledge, attitude, and practice (Fig. 3).

### Gender-responsiveness of AMR grey documents

We analyzed 30 grey documents, including policy, NAP guidelines, and reports. We explored whether documents addressed AMR and the gender-responsiveness of AMR policy documents, and highlighted existing policy gaps (Table 3). Both countries have demonstrated a gradual approach to AMR policy development over the past decade. Bangladesh and Sri Lanka first developed an AMR-focused National Action Plan (2017–2022), followed by AMR Surveillance strategies and a strategic plan that primarily focused on multisectoral coordination under the One Health Framework. It also aligned with the WHO’s global AMR commitments. Among the documents, 22 (Bangladesh: 12, Sri Lanka: 10) addressed AMR, and 8 documents did not address AMR, however, all of the documents were gender-blind (ignored gender norms, and roles, often reinforced gender-based discrimination, and ignored resource allocation for men and women). Most documents considered treating everyone the same, based on the principle of being “fair” (Additional File 4, 5 & 6).

A comparative analysis of policy documents (Additional Files 4 & 5) revealed that all reviewed AMR policies from both countries scored as ‘gender-blind,’ lacking specific provisions for gender-disaggregated surveillance, gender-responsive interventions, or acknowledgment of how.

gender norms shape AMR patterns (see Table 3 for country comparison).

**Table 3:** Comparative summary of grey documents across Bangladesh and Sri Lanka

Country	Total Documents reviewed	AMR addressed	Gender-responsiveness level	Identified Gaps
Bangladesh	15	12	2 (Gender-blind)	No gender and equity-focused action or indicator available
Sri Lanka	15	10	2 (Gender-blind)	No gender and equity-focused action or indicator available

In the subsequent parts of this paper, we demonstrate key findings of the review that integrate the evidence from both peer-reviewed and grey literature to illustrate how gender, equity, and other intersectional factors such as occupation, financial condition, educational status, age, and anatomical structure, influence AMR. Here, the findings are organized into six key themes: (i) Gender-based knowledge, attitude, and practice regarding AMR (ii) Gender disparities in non-recommended antibiotic use and sources (iii) Influence of gender roles and norms in AMR (iv) Gender-based vulnerability to infection and exposure to AMR (v) Role of Healthcare Providers and Prescription Patterns (vi) Drug shop owners’ role and challenges in AMR. To strengthen transparency and thematic coherence, the six emergent themes were explicitly mapped to the five research questions presented in Table 1. Themes 1 to 4 address gender roles, norms, and equity as they influence knowledge, attitudes, practices, and intersectional disparities related to AMR, which aligns with research questions 1 and 2. Themes 5 and 6 reflect the gendered perspectives of both formal and informal healthcare providers and their influence on treatment-seeking and antibiotic use (research questions 3 and 4). Finally, the policy document analysis corresponds to research question 5 by examining the extent to which national AMR policies in Bangladesh and Sri Lanka incorporate gender and equity considerations.

### Gender-based knowledge, attitude, and practice regarding AMR

This theme explored and synthesized evidence on gender-based differences in knowledge, attitude, and practice regarding antimicrobial resistance, and our review revealed that gender-based KAP becomes more complicated when these dimensions intersect other intersectional factors like education, geographic location, and socio-economic status, gender, poverty, etc.

The term “knowledge, attitude, and practice of AMR” refers to an awareness of antibiotic sources (prescription and non-prescription), understanding of AMR, adherence with antibiotic treatment, and barriers to recommended use [28–30].

In Bangladesh, six papers reported that men had better knowledge of AMR concepts and practice than women, especially among university students, veterinary professionals, and parents [28, 31–35]. Poor training facilities and reliance on informal healthcare providers were responsible for knowledge differences and awareness of AMR among males, especially among the male-dominated professions (pharmacy, medicine selling, and poultry farming) [36–40].

Other studies reported that women were mainly responsible for taking care of their children as well as the other sick family members, which helps them to acquire more knowledge through frequent visit in healthcare sectors and conversations with healthcare providers. This led to women knowing more about antibiotics and AMR than men [30, 32, 34, 41–43]. The level of education also played a significant role in knowing about AMR. Mothers with higher education were 1.14 times more likely to access information related to antibiotics compared to mothers with primary education [42]. On the other hand, urban women had more access to healthcare services and information than rural women, especially women from low socio-economic backgrounds [42]. In the veterinary sector, female farmers had less access to knowledge and awareness campaigns than men; as a result, male farmers showed more knowledge and appropriate practice than women farmers [33].

In Sri Lanka, women who had access to the internet and local drug shop owners had a better understanding of antibiotics and AMR than men [44–46], although no gender difference was seen among students [44, 47, 48]. Antibiotic use without any recommendation was similar across genders and the status of education was more influential than gender for it [47, 49].

From our document review of grey articles, we explored AMR-related national policies and guidelines that did not focus on gender-responsive awareness, communication, and education programs. For example, AMR National action plans from Bangladesh and Sri Lanka emphasized awareness, advocacy, and equity, but these did not apply a gender lens and did not consider different messaging for women, men, caregivers, or different vulnerable groups [19–22] (Additional File 4, 5 & 6).

### Gender disparities in non-recommended antibiotic use and sources

This theme identified and synthesized evidence on non-recommended use of antimicrobials, sources among men and women.

We found mixed results in reporting the practice of non-recommended antibiotic use without prescription between men and women. Figure 3 demonstrates that the majority of the articles under this theme were retrieved from Bangladesh (*n* = 13), while we identified four articles from Sri Lanka. In Bangladesh, this practice contributed to AMR among both men and women [50–53], and they relied mostly on local pharmacies for antibiotics [51, 54]. Motivating factors for antibiotic use without recommendation were different for the groups; men tended to use non-recommended antibiotics because of previous experience, social networks, and familiarity with medicine [50–53], while women were guided by cultural norms and healthcare barriers, including lack of proper knowledge [33, 53–55]. Additionally, several studies identified that the rate of buying antibiotics without the recommendation of any professional healthcare provider from the nearby local drug shop or using leftover medicines was higher among women (77%), and was driven by financial constraints, caregiving responsibilities, and healthcare access barriers, especially during the COVID-19 pandemic [33, 34, 54, 56]. Sometimes, women also buy antibiotics for their children without a physician’s prescription [43]. One study found that there was no significant gender association with AMR [28]. However, antibiotic use without any recommendation from professional healthcare providers was more prominent among men because of their autonomy, flexibility to purchase incomplete courses from nearby local drug shops, and taking leftover medicine [34, 41, 52]. In contrast, some studies revealed that male patients were more likely to complete full doses of antibiotics, while women tended to take leftover medicine [43]. In livestock Bangladesh, only 11% of people used a prescription for purchasing antibiotics. Men were more likely to buy medicine from shops than female farmers [33, 57].

In Sri Lanka, the use of antibiotics without any recommended or medically appropriate use of medicines was common in both men and women, but among university students, non-recommended medicine use was higher among male students than female; women were more likely to stop taking antibiotics before completion of the full course [48, 58–60].

Despite significant findings on gender disparities in barriers to recommended antibiotic use and informal providers’ role regarding this matter, we identified several gaps in evidence from Bangladesh and Sri Lanka (Additional Files 4, 5 & 6). For instance, Bangladesh Drug and Cosmetic Act 2023, Bangladesh’s National Drug Policy 2016 and AMR Surveillance Strategy 2020–2025 did not strongly emphasize on informal drug sellers who, in most of the times acted as first point of contact with the health system specially for vulnerable groups (women living in remote areas, women having limited autonomy and financial dependency) [20, 61, 62]. Similarly, the National Strategic Plan for combating AMR 2023–2028, the Sri Lanka Medical Association (SLMA) Antimicrobial Guideline 2024, and the Empirical and prophylactic use of antimicrobials- National guideline 2024 did not mention specifics [22, 63, 64].

### Influence of gender roles and norms in AMR

This review identified and synthesized evidence on how gender roles and norms shape AMR in different ways, such as the division of labor between men and women, autonomy in decision-making, financial dependency, and healthcare access. This review identified a total of 11 studies from Bangladesh (*n* = 9) and Sri Lanka (*n* = 2) on this theme **(**Fig. 3**).**

#### Decision-making power, financial dependency, and access to healthcare

In terms of healthcare decisions, women often relied on their family or community, with limited autonomy and financial independence, whereas men displayed greater autonomy in Bangladesh [17, 41]. While men and women visited urban medicine shops in almost equal proportions, women were underrepresented in rural areas, highlighting the influence of gender norms, such as restricted mobility and limited decision-making autonomy in healthcare access [57]. In Sri Lanka, no study has been found that relates the decision-making power, financial dependency, and access to healthcare regarding AMR.

These findings reflect several structural barriers that compromise human health rights. For example, women frequently experienced limited mobility and autonomy in healthcare access, which increased reliance on nearby informal providers, limited healthcare infrastructure and services, especially in rural contexts, and financial dependency on family members for availing health services [41, 54, 57, 58, 60]. These structural barriers indicate a breach of core principles of health-related rights such as non-discrimination, participation, and empowerment as outlined by WHO [25]. Although, human health right-based lens dimension is unexplored in the reviewed article.

Policy documents from both countries significantly overlooked the gender dimension of decision-making power, financial dependency, and health-care access. In Bangladesh, Standard Treatment Guidelines (2021), National Action Plan (2017–2022), National surveillance strategy (2020–2025), and National Antimicrobial Stewardship Framework did not mention gender-based reality of healthcare access, like limited autonomy and financial dependency, lack of information regarding healthcare service became a central point for their non-recommended antibiotic use [19, 20, 65, 66] Similarly, Sri Lanka’s National Strategic Plan for combating AMR (2017–2022, 2023–2028), National Action Plan for Health Security of Sri Lanka 2019–2023, Empirical and prophylactic use of antimicrobials- National guideline (2016, 2024), did not focus on gender norms impact on healthcare seeking behavior and access [21, 22, 64, 67, 68].

#### Distribution of labor and practices

We identified relevant studies from Bangladesh (*n* = 5) and Sri Lanka (*n* = 2) **(**Fig. 3**)**. Gender norms and disparities in labor distribution were closely correlated with AMR risks [29, 52, 69]. In commercial settings of poultry farming, live bird markets, or agriculture, men were exposed to AMR risks through frequent contact with animal blood and waste, and minimal use of protective equipment and handwashing, and higher usage of antimicrobials [52, 69].

In contrast, women were exposed through household farming and childcare [69, 70]. In rural areas, engaging in both the activities, keeping animals inside living spaces, and multitasking without proper handwashing were contributing factors for AMR [70]. Furthermore, inappropriate waste management and the use of poultry manure as fertilizer caused environmental contamination and increased the risk of AMR [70].

Among female sex workers (FSW), sexually transmitted infections (STIs) and Neisseria gonorrhoeae culture tests showed higher resistance to Cefixime and Azithromycin among residence-based FSWs than street-based FSWs, suggesting possible differences in exposure [71].

In Sri Lanka, men were also found to be at risk through livestock farming, and they showed higher levels of resistance to antibiotics [72]. Additionally, at the household level, male parents or male caregivers showed lower capability compared to females for practicing oral pediatric antibiotic suspensions (OPAS) [73].

Despite gender-based labor, such as women’s role as caregivers, household farming, and men’s involvement in commercial poultry and livestock, which influenced AMR exposure in significant ways but these aspects are missing in documents from both countries. For instance, the Bangladesh National Action Plan on AMR (2027 − 2022), Strategic Framework and Action Plan for the Application of a One Health Approach in Bangladesh (2017–2021), National Antimicrobial Resistance (AMR) Surveillance Strategy of Bangladesh 2020–2025, National Environment Policy 2018 [74], as well as Sri Lanka’s National Strategic Plan for combating AMR (2017–2022, 2023–2028), National Action Plan for Health Security of Sri Lanka 2019–2023, National Occupational Safety and Health Policy emphasized multisectoral coordination, infection, prevention but did not have targeted strategies to mitigate vulnerabilities based on distribution of labors and practices [19–22, 67, 75, 76].

### Gender-based vulnerability to infection and exposure to AMR

This review identified and synthesized seven and eight articles, respectively, from the Bangladesh and Sri Lanka contexts that directly and indirectly focused on the intersection between gender and biological disease susceptibility and vulnerability regarding AMR **(**Fig. 3**)**.

In Bangladesh, women were found more susceptible to UTIs, which was primarily driven by anatomical structure e.g., shorter urethras than men, undernutrition, primiparity, and linked to low paternal education, while men between 10 and 60 years of age were more likely to test positive for Multidrug Resistance (MDR) pathogens [77–80]. Align with that, one article also found gender-based differences regarding gene distribution in bacteria isolates, for instance, female patients carry higher blaNDM-1 gene which cause resistance to strong antibiotics while male patients more often carried blaSHV-11 genes which contribute to β-lactam antibiotics as well as some articles found higher multidrug-resistant organisms (MDROs) genes among women for instance and no gender difference was found in antimicrobial patterns among children under 5 [80–83].

In Sri Lanka, pregnant, post-partum, and unemployed women were prone to vaginal colonization of multidrug-resistant organisms (MDRO) [84]. Regarding antiretroviral therapy (ART) for Human immunodeficiency virus, pretreatment drug resistance was found to be higher in women than in men [85]. Additionally, two studies highlighted gender with age -based differences in antimicrobial susceptibility among UTI patients, where men and adults have lower susceptibility to first-line antibiotics [86, 87]. Males have more susceptibility to methicillin-resistant Staphylococcus aureus (MRSA) than females [88]. In contrast, one study observed no significant gender difference in community or hospital-acquired MRSA [89]. Additionally, newborns also showed susceptibility to MDRO, which is caused by breastfeeding and skin-to-skin contact [86, 90, 91] with women and young people.

Although there is clear evidence on gender-based vulnerability, national policies, NAP, strategic plan in Bangladesh such as, the National Action Plan on AMR [19, 20], National Patient Safety Strategic Plan in Bangladesh 2018 [92], Digital Health Strategy 2023–2027 of Bangladesh [93] did not have evidence or measures related to gender specific infection, prevention, and control. Additionally, Sri Lanka’s Strategic Plan for Combating AMR (2017–2022, 2023–2028), National Antimicrobial Stewardship Guideline for Healthcare Institutions [94], National Health Strategic Master Plan 2016–2025 [95], National Health Policy 2016–2025 [96], National Policy on Health Information [97], did not emphasize social or biological factors related to gender-based disease exposure, infection, prevention, control, data indicator, gender-specific information system and vulnerability [21, 22].

### Role of healthcare providers and prescription patterns

This theme identified and synthesized the evidence on gender-related disparities in antibiotic prescription patterns in both countries. In Bangladesh, more than one-fifth of patients were recommended diagnostic tests for disease identification before prescribing an antibiotic, about 82% of cases were prescribed an antibiotic based on the physician’s presumptive diagnosis, and only 18% antibiotics were prescribed based on culture and sensitivity tests [98]. The rate of prescribing antibiotics by the healthcare providers was higher among men than women [98, 99]. Women were more likely to present a prescription when purchasing antibiotics at the drug shops, whereas men were more often to buy antibiotics without a prescription [57].

### Drug shop owners’ role and challenges in AMR

This theme explored and synthesized the evidence on drug shop owners and their challenges. Drug shops (unlicensed pharmacies) **s**erve as the main gatekeepers between doctors and patients but face dilemmas selling antibiotics to their customers without prescriptions and this is evident from both community people and service providers’ perspectives [37, 41]. Some drug sellers were forced to sell antibiotics without a prescription due to customer demand and in order not to lose any customers and the income. Additionally, due to a lack of proper knowledge, some of them believe that it was not their responsibility to follow the rules of antibiotic sales [37, 41]. Usually, some pharmacists provide basic instructions about antibiotic consumption, but they were unmotivated to consult with their customers when they did not follow the recommended courses of treatment. Furthermore, cultural constraints restricted the number of women in pharmacist positions, which further narrowed the field for women in healthcare delivery [37]. In Sri Lanka, it was found that there was no significant association between gender and prescribing pattern [100].

Despite these findings, national AMR policies from Bangladesh and Sri Lanka did not focus on these gender dimensions, like disparities in prescription patterns and barriers to recommended antibiotic use. For example, the Bangladesh Drug and Cosmetic Act 2023 [61], Standard Treatment Guidelines (STG) Bangladesh on Antibiotic Use 2021 [65], National Drug Policy Bangladesh 2016 [62], SLMA Antimicrobial Guideline Sri Lanka [63], National Medicines Regulatory Authority Act, No. 5 of 2015 Sri Lanka [101], Implementation of AMR prescription chart in healthcare, Sri Lanka [102], Implementation of Aware classification of AMR in Sri Lanka [103] and National Antimicrobial Stewardship Guideline for Healthcare Institutions [94] mainly focused on promoting rational use of antibiotic, institutional accountability, prescription based antibiotic selling. But these policies did not recognize the gender aspects of prescribing practice, gender-based barriers to recommended antibiotic use, and overlooked informal drug sellers, including gender-responsive or gender and equity-based training among formal and informal healthcare providers (Additional File 4, 5 & 6).

## Discussion

This scoping review from Bangladesh and Sri Lanka revealed that AMR is significantly shaped by gender as well as strongly influenced by the intersection with multiple axes of inequity such as gender norms, education, poverty, occupation, geographic location, healthcare access and autonomy, cultural norms and family roles, socio-economic status, including policy inclusion and implementation. This review revealed that AMR needs to be understood from multiple axes, compared to single axis approaches, such as gender is not the only key determinant. Single-axis analysis revealed gender inequalities in AMR exposure and access to care, whereas, intersectional analysis showed interactions of gender with other social determinants to result in compounded vulnerabilities. Using the example of a single-axis gender analysis, it might be concluded that women tend to take antibiotics without prescription more frequently. Nevertheless, an intersectional prism shows that this trend is especially acute among low-income rural and women with limited mobility and financial independence, whose structural restrictions to access to formal healthcare push them to informal providers. In the same vein, although the rate of non-recommended use of antibiotics might be higher among men, intersectional analysis demonstrates that this is particularly pronounced among men engaged in occupations such as livestock farming, where occupational exposure and easy access to antimicrobials intersect. These illustrations indicate that gender only cannot be used to explain the dynamics of AMR without interacting social determinants.

The intersectional lens used throughout this scoping review (60 peer-reviewed articles and 30 documents) unpacks the interaction between gender and other intersectional inequalities such as socio-demographic inequalities, occupation, and geography, which play a significant role in shaping AMR knowledge, attitude and practices, health outcomes and access, non-prescribed antibiotic use, role of formal and informal healthcare providers, including socially determined vulnerabilities in Bangladesh and Sri Lanka. For example, in Bangladesh, rural women often use antibiotics without a doctor’s prescription because of their financial crisis, dependency, and healthcare access. While in Sri Lanka, young men at universities were more likely not to finish their medicine, mostly because they could easily buy antibiotics from local shops and felt confident making their own decisions. These examples illustrate how intersectionality deepens our analysis by identifying which subgroups within “women” or “men” experience heightened risk, and why policy responses must differentiate across all the groups. Gender and other intersectional factors (e.g., education, poverty, occupation, geographic location, healthcare access and autonomy, cultural norms, and family roles) are the key determinants in shaping health outcomes and access, inherently influencing the health system [11, 104, 105].

Our scoping review findings are consistent with the evidence from other low-and middle-income countries (LMICs), such as Southeast Asia, and Sub-Saharan Africa, although those gendered AMR patterns in Bangladesh and Sri Lanka are driven by context-specific structural factors such as poor informal health markets, a lack of primary healthcare coverage and ingrained gender norms around antibiotic access and use. Informal drug sellers in such environments frequently serve as the first point of care, especially among women whose access to care is limited by their limited mobility, caregiving duties and financial reliance, a trend also observed in India, Kenya, Nigeria and Tanzania [106, 107].

A systematic study also revealed that, in Africa, women are vulnerable and frequently self-medicate due to the cost and mobility barrier than in other regions [107]. However, this pattern is even more pronounced in Bangladesh and Sri Lanka because primary care systems in many rural settings remain fragmented, under-resourced, and constraining gendered decision-making norms make women heavily reliant on informal drug sellers as the first point of contact. These dynamics appear to be even stronger due to extremely fragmented systems of primary healthcare and the informal pharmaceutical markets. These factors increase the level of dependence on over-the-counter antibiotics and legitimize the use of non-recommended drugs [8, 17, 39, 49, 106].

Simultaneously, gendered norms of decision-making interplay with occupational risk factors to generate disparate AMR risks, men often are more vulnerable to AMR in sectors such as livestock, agriculture, aquaculture, and transport sectors which is similar to findings from India, Nigeria, and Uganda, but differs in its scale in Bangladesh where large informal agricultural and poultry markets increase daily exposure to contaminated waste and antimicrobial residues [33, 106, 108, 109].

Additionally, aligning with our findings, one study focused on LMICs revealed that AMR affects different groups differently; in particular, certain marginalized communities are more affected than others, primarily due to social and structural determinants of health. However, interventions and actions regarding AMR frequently neglect the gender and equity dimensions and overlook the larger societal picture underlying the root causes of AMR risk in South Asia, in comparison with certain African and Latin American LMICs where community health worker initiatives partly mitigate informal access to antibiotics [107, 109].

So, for the sustainable development in the overall health system along with AMR containment, gathering gender-disaggregated data, analysis, and fostering equal access to healthcare are crucial [110]. Due to the contextual variations, AMR policy responses in Bangladesh and Sri Lanka cannot rely solely on global recommendations but must address local structural factors, including informal health markets, health service accessibility, and gendered decision-making norms. These and other issues are discussed with implications for designing and implementing more gender-responsive policies and practices to mitigate AMR risk.

From this review we observed that AMR knowledge depends on multiple dimensions, for example, women generally gain knowledge of antibiotics and AMR because of their caregiving roles, necessitating frequent interactions with the healthcare system, both from Bangladesh and Sri Lanka [42–44], as is also observed in a study from South-east Asia [111], and studies conducted in Italy and Thailand [112, 113].

Gender-based disparities in AMR knowledge and practice may differ according to context. For example, while some studies show that men with higher education or involvement in the veterinary profession had higher knowledge of AMR [31], some other studies show no gender variation [44, 46, 48]. In those instances, context (e.g., education and socioeconomic status) may become more significant [111–113].

In terms of practice, other studies beyond Bangladesh and Sri Lanka also observed that men often use non-recommended antibiotics, meaning they use antibiotics without a professional prescription due to their familiarity with them, ease of access, and relationships with medicine sellers [50, 60, 114–117]. However, conflicting data indicate that in some situations, women may use non-recommended antibiotics at higher rates due to their caregiving obligations and financial limitations [118].

Additionally, in the Bangladesh context, time is a key factor that critically influences health-seeking behavior for people like daily laborers who do not have the luxury to lose daily wages due to prolonged waiting times at clinics or hospitals, so they mostly rely on unregulated retail drug sellers as a practical source of treatment [117]. This suggests the necessity for focused education programs and contextually appropriate policy that use community health initiatives, including interventions that address underlying structural inequalities that prevent people from using antibiotics in recommended ways to address risky behaviors among consumers.

Additionally, strict laws, legislation and regulation monitoring systems need to be established to restrict the non-prescribed over-the-counter medicine sales by local drug sellers. However, evidence from Mexico suggests that reinforcement of laws alone cannot tackle non-prescribed medicine sales if the root causes of the barriers to recommended antibiotic use are not addressed [119]. In Bangladesh, similar patterns were already observed in our review. Even if there is existing legislation against non-prescribed antibiotic sales but due to structural inequalities including low income, ease of relationship with medicine seller, trust, lack of knowledge, prolonged waiting time, gender-based norms, roles and responsibilities, geographical location, and lack of proper healthcare access in nearer proximity drive towards informal drug sellers as their primary treatment source. As a consequence, informal healthcare providers often sell antibiotics driven by patient demand and self-financial benefits [37, 41].

Findings also reveal the role of gender norms and relations on exposure to drug-resistant diseases due to occupational hazards [69, 120]. For example, men are more likely to work in occupations that include direct contact with resistant diseases, such as farming, livestock raising, and fishing, where the use of antibiotics is common [58, 69]. On the other hand, women are more vulnerable to exposure to resistant infections since they are responsible for kitchen chores, providing care, engaging with healthcare facilities frequently, and having less healthcare decision-making power [41]. The reviewed literature and polices identified a significant evidence gap between AMR and feminized factory sectors such as ready-made garments in Bangladesh (RMG), while several studies have already highlighted female workers’ crowded work environment, limited healthcare access, and occupational pressures, which create health issues [121, 122]. Additionally, one ethnographic study revealed a female street hawker suffering from UTIs and dehydration frequently due to limited access to toilets and basic water, sanitation, and hygiene facilities [117]. To identify AMR risks among this group of female workers, targeted policy, feminized factory-focused research is essential.

Although reviewing literature from both countries showed that women faced socioeconomic and cultural barriers to access health services, the current policies do not specifically address gender issues, even though gender has a significant role in determining antibiotic use, illness exposure, and healthcare access [8, 123]. According to the WHO, these structural barriers reflect fundamental health-rights violations whose principles are non-discrimination, participation, and empowerment [25]. This study has also reiterated the role of biological differences in acquiring resistant infections and ultimately AMR, e.g., females have a greater incidence of urinary tract infections (UTIs) due to anatomical vulnerability, while males are more vulnerable to having bloodstream infections and MRSA carriage, possibly as a result of hormonal variations and poorer hygiene compliance [124]. Additionally, age affects AMR risk; younger women, pregnant women, and older men are more likely to get UTIs [125, 126]. Sexual behavior also plays a role in AMR risk; female sex workers are more likely to have drug-resistant Neisseria gonorrhoeae infections, and females are more likely to have asymptomatic gonorrhea [71, 104].

The results of this paper have several benefits for both the policymakers and the programme designers. There is a variation of knowledge, attitude, and practice among males and females due to their socioeconomic status, occupation, and gender roles and norms in society, yet research articles are lacking. A book from Bangladesh, *“Poverty*,* Gender and Health in the Slums of Bangladesh”*, revealed that poverty acts as a constant structural barrier to healthcare access, especially in urban informal settlements. This book portrays how financial insecurity, gender-based cultural norms, and roles shape health-seeking behavior, especially for women [114]. The reviewed articles did not focus on a health-rights-based approach, so future research should focus on human-rights-based lenses to produce equitable, gender-responsive, and evidence-based recommendations for upcoming AMR national policy formulation, implementation, and monitoring. In addition, this review identifies other research priorities such as intersectional gender analysis across the human, animal, and environmental sectors, the need to explore the gender dimension of AMR use, access, and policy implementation research to understand gaps in multisectoral coordination, including gender and equity integrations. This is critical, as currently all the national AMR documents we reviewed were gender-blind in both countries. Thus, the use of the WHO Gender Responsive Assessment Scale, intersectional lens, gender-responsive indicators, including a monitoring and accountability system in all relevant national-level policies, strategies, and action plans, will significantly strengthen upcoming National AMR policies and interventions in a gender-responsive and equitable way.

This study explored the intersectionality framework to explore how social and structural determinants, such as gender, age, and disability, shape AMR. But we were limited by the scarcity of significant gender-disaggregated data in the AMR research field, which constrains our understanding of different vulnerable groups, and this gap hinders health equity and accountability in AMR-specific interventions. Furthermore, a comparison of our findings with international literature revealed that gathering proper intersectional data is a common difficulty. There are still gaps in surveillance systems across settings since they are not yet structured to capture this complexity. We contribute to filling this knowledge gap with additional information and data, enabling policymakers, program implementers, and academia to make more sustainable decisions regarding combating AMR through context-specific strategies, policies, and interventions. Based on the above, recommendations include the use of an intersectional gender lens for targeted context-specific and gender-sensitive awareness campaigns and intervention, training, and education programs to be incorporated among formal and informal healthcare providers as well as within community people. For example, implementing biosecurity measures for men who are involved with agriculture, poultry farming, aquaculture and other risky professions; awareness campaigns for women about safety in their caregiving role; developing concrete roadmaps for implementation with strong political commitment; strengthening the laws or regulations for regulating local medicine sellers and pharmaceutical companies to ensure evidence-based and ethical information circulation including responsible marketing of recommended use of antibiotics and reduction of non-recommended antibiotic use across genders; and improving access to healthcare services for women, both geographically and financially. In addition, these regulation and awareness initiatives must also cover animal health as well as the environment from a one health dimension.

### Limitations and strengths

In this scoping review of gender equity in the AMR situation in Bangladesh and Sri Lanka, there is a lack of papers on marginalized groups such as LGBTQIA+ individuals, persons with disabilities, and feminized factory work that explain the gender lens for AMR. Even included papers only represent binary gender categories, and in the gender lens analysis, they have information gaps. So, we recognize that our analyses are limited due to a lack of relevant data. Additionally, due to restricted access to some of the databases, the search may not be comprehensive, and limited access to grey literature sources may not include some policies as those policies are unavailable online. Also, only English-language and open-access articles were considered for inclusion in this review. However, we will acknowledge this as a limitation, as it may have excluded relevant regional-language resources and may cause potential selection bias. Additionally, this scoping review has the possibility of temporal bias due to the overrepresentation of current studies, due to recent growth in interest around the issue, and geographical limitations as this review only focused on two Southeast Asian countries.

Despite these limitations, this study is the first comprehensive scoping review on gender and equity within AMR in Bangladesh and Sri Lanka, as well as the Southeast Asia region. Additionally, this review uses a robust intersectional analytical approach for analyzing evidence from both peer-reviewed article and grey documents. Our findings are directly policy-relevant, providing a foundation for gender-responsive AMR action planning and surveillance in the region. For strong methodological adherence, this review followed PRISMA-ScR guidelines.

## Conclusion

This review reveals the evidence and critical gaps that gender disparities, along with intersectional factors, significantly influence AMR vulnerabilities in Bangladesh and Sri Lanka. Socio-cultural norms and roles, financial dependency, and limited autonomy hindered women’s timely formal healthcare access, which influences them to use non-recommended antimicrobials from the closest and cheapest local drug stores and increase their AMR exposure. Meanwhile, men’s occupational roles, past experience, peer influence, and easy access increase their AMR exposure and non-recommended antimicrobial use. This review identified significant gender gaps and equity-focused AMR research, gender-disaggregated AMR data, and integration of gender and equity dimensions in policy documents. So, further research is needed to explore how gender dynamics influence healthcare-seeking practice and AMR exposure within the household and workplace, underlying root causes of inequities relating to AMR exposure, access, and use. Addressing these gaps will help to develop gender-responsive policies along with improved healthcare structures, which are essential to mitigate AMR risks and promote equitable health outcomes. Incorporating gender-responsive approaches into research, policies, and healthcare practices is essential to identify the discrepancies and implement the interventions effectively for sustainable solutions to the global AMR epidemic. Research and action must go beyond one health to include the intersections of human health, animal health, and environmental health. Policymakers should consider gender and equity-focused indicators in surveillance systems, and Gender-responsiveness regarding AMR Naps, and strategies.

## Supplementary Information

Additional file 1.

Additional file 2.

Additional file 3.

Additional file 4.

Additional file 5.

Additional file 6.

Additional file 7.

Additional file 8.

Additional file 9.

Additional file 10.

Additional file 11.

Additional file 12.

## Data Availability

All raw data is stored in the institutional repository against the corresponding author’s organizational email for 5 years following the policy, and is available upon reasonable request.

## Abbreviations

AMR: Antimicrobial Resistance
LMICs: Low- and Middle-Income Countries
WHO: World Health Organization
NAP: National Action Plan
WASH: Water, Sanitation, and Hygiene
UTIs: Urinary Tract Infections
PCC: Population, Concept, Context
KAP: Knowledge, Attitude, and Practice
HCPs: Healthcare Providers
MRSA: Methicillin-Resistant *Staphylococcus aureus*
MDROs: Multidrug-Resistant Organisms
FSW: Female Sex Worker
OPAS: Oral Pediatric Antibiotic Suspensions
STIs: Sexually Transmitted Infections
ART: Antiretroviral Therapy
RMG: Ready-Made Garments
STG: Standard Treatment Guidelines
OTC: Over-the-Counter
