# Supplementary material for: Gender and equity within antimicrobial resistance: a scoping review of evidence and policy in Bangladesh and Sri Lanka
Source: BMC Public Health. 2026 Jun 4;26:2313. doi: 10.1186/s12889-026-27842-y (PMC13455460; doi:10.1186/s12889-026-27842-y)
Supplement: Supplementary file 1 [file 12889_2026_27842_MOESM10_ESM.docx]

## Sheet: Bangladesh

Author name	Year	Title	Decision	Reason for Exclusion
M. Abd El Ghany, N. Fouz and G. A. Hill-Cawthorne	2020	Human movement and transmission of antimicrobial-resistant bacteria	Excluded	Review paper
I. Ahmed, R. King, S. Akter, R. Akter and V. R. Aggarwal	2023	Determinants of antibiotic self-medication: A systematic review and meta-analysis	Excluded	Review paper
I. Ahmed, M. B. Rabbi and S. Sultana	2019	Antibiotic resistance in Bangladesh: A systematic review	Excluded	Review paper
S. Ahmed, P. Korpe, T. Ahmed, M. J. Chisti and A. S. G. Faruque	2018	Burden and Risk Factors of Antimicrobial Use in Children Less Than 5 Years of Age with Diarrheal Illness in Rural Bangladesh	Excluded	No gender segregated information
S. M. Ahmed, A. M. Adams, M. Chowdhury and A. Bhuiya	2000	Gender, socioeconomic development and health-seeking behaviour in Bangladesh	Excluded	Not AMR related
Z. Akhtar, S. Mah-E-Muneer, M. M. Rashid, M. S. Ahmed, M. A. Islam, S. Chowdhury, Z. Khan, M. Z. Hassan, K. Islam and S. Parveen	2021	Antibiotics use and its knowledge in the community: a mobile phone survey during the COVID-19 pandemic in Bangladesh	Excluded	No gender segregated information
Akter, shikha	2016	Prescription Pattern of Antibiotic among the Specialist Doctors in Different area of Bangladesh	Excluded	Not peer reviewed
S. F. U. Akter, M. F. A. Rani, M. Y. Rathor, M. A. B. M. Aris, M. A. Jabbar and S. K. Mazumder	2012	Hospital physicians’ drugs prescription adherence to the essential drugs list of Bangladesh	Excluded	Timeframe
S. Al Sanjee, M. Rahman, M. E. Karim and U. S. Sigma	2020	Prevalence, associated risk factors and antibiotic resistance pattern of bacterial uropathogens among pregnant women in a tertiary care hospital of Bangladesh	Excluded	No gender segregated information
G. Allwell-Brown, L. Hussain-Alkhateeb, M. O. Sewe, F. E. Kitutu, S. Strömdahl, A. Mårtensson and E. W. Johansson	2021	Determinants of trends in reported antibiotic use among sick children under five years of age across low-income and middle-income countries in 2005–17: A systematic analysis of user characteristics based on 132 national surveys from 73 countries	Excluded	No gender segregated information
S. Ashreen, A. Ahmed, N. Hasan, W. Akhtar and M. M. Hossain	2019	Isolation and identification of gram-negative bacteria from oral cancer site infections and study of their antibiotic resistance pattern	Excluded	No relevent information
O. A. Asif, M. A. Habib and M. A. Islam	2020	ASSESSMENT OF RATIONAL USE OF ANTIBIOTICS IN TWO TERTIARY HOSPITAL	Excluded	No gender segregated information
A. Baldi, S. Braat, M. Imrul Hasan, C. Bennett, M. Barrios, N. Jones, G. Moir-Meyer, I. Abdul Azeez, S. Wilcox, M. Saiful Alam Bhuiyan, R. Ataide, D. Clucas, L. C. Harrison, S. E. Arifeen, R. Bowden, B. A. Biggs, A. Jex and S. R. Pasricha	2024	Community use of oral antibiotics transiently reprofiles the intestinal microbiome in young Bangladeshi children	Excluded	No relevent information
S. Banu, M. T. Rahman, S. Ahmed, R. Khatun, S. S. Ferdous, B. Hosen, M. M. Rahman, T. Ahmed, J. S. Cavanaugh and J. D. Heffelfinger	2017	Multidrug-resistant tuberculosis in Bangladesh: results from a sentinel surveillance system	Excluded	No relevent information
Baral, Poulomi, Afnan, Nafisa, Ahmad Zahra, Maftuha, Akter, Baby, Rabia Prapti, Shek, Muazzam Hossan, Mohammed, Haque, Fahim Kabir Monjurul.	2024	Bacteriological analysis and antibiotic resistance in patients with diabetic foot ulcers in Dhaka	Excluded	No relevent information
A. Begum, M. S. Bari, F. R. Chowdhury, N. Ahmed and K. A. R. Sayeed	2015	Pattern of anti-microbial sensitivity and resistance against Salmonella Species in a Tertiary Hospital in Dhaka	Excluded	No relevent information
H. Begum, M. M. Rowshan, S. Khanom, S. Haque, F. Afroze and A. N. Dina	2015	Prescribing Pattern in Outpatient Departments of Two Tertiary Care Teaching Hospitals in Dhaka	Excluded	Not AMR related
Y. A. Begum, K. A. Talukder, I. J. Azmi, M. Shahnaij, A. Sheikh, S. Sharmin, A. M. Svennerholm and F. Qadri	2016	Resistance Pattern and Molecular Characterization of Enterotoxigenic Escherichia coli (ETEC) Strains Isolated in Bangladesh	Excluded	No gender segregated information
T. Belkina	2017	Non-prescribed antibiotic use in some developing countries and its association with drug resistance	Excluded	Not country specific
Bhuiyan, Md Salman Shah.	2017	A Cross Sectional Study of Prescribing Patterns for Acute Respiratory Infections in Public & Private Hospitals in Dhaka Metropolis	Excluded	No relevent information
A. S. Bonna, S. R. Pavel, J. Ferdous, S. A. Khan and M. Ali	2022	Antibiotic resistance: An increasingly threatening but neglected public health challenge in Bangladesh	Excluded	No relevent information
K. Boone, S. K. Morris, S. Doshi, J. Black, M. Mohsin, T. Ahmed, A. Al Mahmud and D. E. Roth	2021	Antimicrobial Prescribing during Infant Hospital Admissions in a Birth Cohort in Dhaka, Bangladesh	Excluded	No gender segregated information
F. Chowdhury, S. Mah-E-Muneer, S. Bollinger, A. Sharma, D. Ahmed, K. Hossain, M. Z. Hassan, M. Rahman, D. Vanderende and D. Sen	2023	Prevalence of colonization with antibiotic-resistant organisms in hospitalized and community individuals in Bangladesh, a phenotypic analysis: findings from the antibiotic resistance in communities and hospitals (ARCH) study	Excluded	No gender segregated information
M. K. Chowdhury, A. A. Siddique, P. K. Sarkar, M. Haque, P. K. Biswas, S. Biswas and H. N. Ahasan	2015	Pattern of antibiotic use in different departments of Dhaka medical college hospital	Excluded	No gender segregated information
M. M. H. Chowdhury, K. Kubra, M. T. Islam, M. M. Rahman and M. E. Mehedy	2015	Indiscriminate uses of antibiotics as a threat to public health demand implementation of effective drug practices and enhancement of public awareness in Bangladesh	Excluded	Full text not available
S. Chowdhury, G. Fournié, D. Blake, J. Henning, P. Conway, M. A. Hoque, S. Ghosh, S. Parveen, P. K. Biswas, Z. Akhtar, K. Islam, M. A. Islam, M. M. Rashid, L. Pelligand, Z. H. Khan, M. Rahman, F. Tomley, N. Debnath and F. Chowdhury	2022	Antibiotic usage practices and its drivers in commercial chicken production in Bangladesh	Excluded	No relevent information
S. Chowdhury, S. Rheman, N. Debnath, J. Delamare-Deboutteville, Z. Akhtar, S. Ghosh, S. Parveen, K. Islam, M. A. Islam, M. M. Rashid, Z. H. Khan, M. Rahman, V. M. Chadag and F. Chowdhury	2022	Antibiotics usage practices in aquaculture in Bangladesh and their associated factors	Excluded	No relevent information
R. Dacombe, I. Bates, M. Bhardwaj, S. Wallis and J. Pulford	2016	Fleming Fund: supporting surveillance capacity for antimicrobial resistance An analysis of approaches to laboratory capacity strengthening for drug resistant infections in low and middle income countries	Excluded	Not country specific
S. Daria and M. R. Islam	2022	Indiscriminate use of antibiotics for COVID-19 treatment in South Asian countries is a threat for future pandemics due to antibiotic resistance	Excluded	Not country specific
S. K. Das, S. Ahmed, F. Ferdous, F. D. Farzana, M. J. Chisti, J. R. Latham, K. A. Talukder, M. Rahman, Y. A. Begum, F. Qadri, A. S. Golam Faruque and T. Ahmed	2013	Etiological diversity of diarrhoeal disease in Bangladesh	Excluded	No relevent information
C. Dasgupta, M. A. Rafi and M. A. Salam	2020	High prevalence of multidrug resistant uropathogens: A recent audit of antimicrobial susceptibility testing from a tertiary care hospital in bangladeshn	Excluded	No gender segregated information
S. K. Datta, T. R. Paul, M. Monwar, A. Khatun, M. Rafiqul Islam, M. Ashraf Ali, R. K. Barman, B. M. Rahman and M. I. I. Wahed	2016	Patterns of prescription and antibiotic use among outpatients in a tertiary care teaching hospital of Bangladesh	Excluded	No gender segregated information
Z. A. Dyson, P. M. Ashton, F. Khanam, A. Chunga Chirambo, M. Shakya, J. E. Meiring, S. Tonks, A. Karkey, C. Msefula, J. D. Clemens, S. J. Dunstan, S. Baker, G. Dougan, V. E. Pitzer, B. Basnyat, F. Qadri, R. S. Heyderman, M. A. Gordon, A. J. Pollard and K. E. Holt	2024	Pathogen diversity and antimicrobial resistance transmission of Salmonella enterica serovars Typhi and Paratyphi A in Bangladesh, Nepal, and Malawi: a genomic epidemiological study	Excluded	No gender segregated information
K. Farjana, H. I. Zahid, M. S. Bhuiya and S. Yesmine	2015	Pattern of antibiotic use and physician s opinion about the resistance against antibiotics used for treating Respiratory Tract Infections (RTIs) in Bangladesh: A cross sectional survey	Excluded	No gender segregated information
K. Fatema, A. S. M. A. Ahsan, L. Barai, F. Ahmed, J. A. Haq and M. O. Faruq	2016	Bacterial profile and their antibiotic resistance in an ICU of Bangladesh: Comparison of four studies from 2004 to 2011	Excluded	No gender segregated information
Garbern, S. C., Chu, T. C., Gainey, M., Kanekar, S. S., Nasrin, S., Qu, K., Barry, M. A., Nelson, E. J., Leung, D. T., Schmid, C. H., Alam, N. H., Levine, A. C.	2021	Multidrug-resistant enteric pathogens in older children and adults with diarrhea in Bangladesh: epidemiology and risk factors	Excluded	No gender segregated information
S. C. Garbern, T. C. Chu, M. Gainey, S. S. Kanekar, S. Nasrin, K. Qu, M. A. Barry, E. J. Nelson, D. T. Leung, C. H. Schmid, N. H. Alam and A. C. Levine	2021	Clinical and socio-environmental determinants of multidrug-resistant vibrio cholerae 01 in older children and adults in Bangladesh	Excluded	No gender segregated information
J. M. C. Gautron, G. Tu Thanh, V. Barasa and G. Voltolina	2021	Using intersectionality to study gender and antimicrobial resistance in low-and middle-income countries	Excluded	Review paper
Z. H. Habib, S. b. Golam Rasul, M. A. Alam, N. N. Bably, I. A. Khan, S. M. S. Rizvi, T. Shirin, A. N. Alam, M. S. Uzzaman and A. S. M. Alamgir	2021	The findings of antimicrobial resistance surveillance in Bangladesh (2016-2020)	Excluded	Not peer reviewed
A. Hafsa, M. Fakruddin, M. A. Hakim and J. D. Sharma	2011	Neonatal bacteremia in a neonatal intensive care unit: Analysis of causative organisms and antimicrobial susceptibility	Excluded	No gender segregated information
D. H. Hamer, G. L. Darmstadt, J. B. Carlin, A. K. Zaidi, K. Yeboah-Antwi, S. K. Saha, P. Ray, A. Narang, E. Mazzi, P. Kumar, A. Kapil, P. M. Jeena, A. Deorari, A. K. Chowdury, A. Bartos, Z. A. Bhutta, Y. Adu-Sarkodie, M. Adhikari, E. Addo-Yobo and M. W. Weber	2015	Etiology of bacteremia in young infants in six countries	Excluded	No gender segregated information
M. Haque	2017	Antimicrobial use, prescribing, and resistance in selected ten selected developing countries: a brief overview	Excluded	Review paper
S. Haque	2017	Association between self-medication behavior and adherence among medical students in Dhaka, Bangladesh	Excluded	No gender segregated information
M. D. Harun, M. R. Haider, A. A. Chowdhury, I. Haque and S. Shrmin	2020	Pattern of antibiotics use and health seeking behavior among low income urban under five children in Bangladesh	Excluded	No gender segregated information
M. G. D. Harun, M. M. U. Anwar, S. A. Sumon, M. Z. Hassan, T. M. Mohona, A. Rahman, S. A. H. M. Abdullah, M. S. Islam, S. C. Kaydos-Daniels and A. R. Styczynski	2022	Rationale and guidance for strengthening infection prevention and control measures and antimicrobial stewardship programs in Bangladesh: A study protocol	Excluded	No gender segregated information
M. M. Hassan, K. B. Amin, M. Ahaduzzaman, M. Alam, M. S. Faruk and I. Uddin	2014	Antimicrobial resistance pattern against E. coli and Salmonella in layer poultry	Excluded	No relevent information
M. M. Hassan, M. A. Kalam, M. A. Alim, S. Shano, M. R. K. Nayem, M. R. Badsha, M. A. Al Mamun, A. Hoque, A. Z. Tanzin and C. Nath	2021	Knowledge, attitude, and practices on antimicrobial use and antimicrobial resistance among commercial poultry farmers in Bangladesh	Excluded	No gender segregated information
M. Z. Hassan, M. R. Monjur, M. A. A. J. Biswas, F. Chowdhury, M. A. H. Kafi, J. Braithwaite, A. Jaffe and N. Homaira	2021	Antibiotic use for acute respiratory infections among under-5 children in Bangladesh: a population-based survey	Excluded	No relevent information
C. Hemlock, S. P. Luby, S. Saha, F. Qamar, J. R. Andrews, S. K. Saha, D. Tamrakar, K. Date, A. T. Longley, D. O. Garrett and I. I. Bogoch	2020	Utilization of Blood Culture in South Asia for the Diagnosis and Treatment of Febrile Illness	Excluded	Not country specific
S. K. Heysell, S. Ahmed, S. S. Ferdous, M. S. Khan, S. M. Rahman, J. Gratz, M. T. Rahman, A. M. Mahmud, E. R. Houpt and S. Banu	2015	Quantitative drug-susceptibility in patients treated for multidrug-resistant tuberculosis in Bangladesh: implications for regimen choice	Excluded	No AMR related information
Y. Hooda, M. S. I. Sajib, H. Rahman, S. P. Luby, J. Bondy-Denomy, M. Santosham, J. R. Andrews, S. K. Saha and S. Saha	2019	Molecular mechanism of azithromycin resistance among typhoidal Salmonella strains in Bangladesh identified through passive pediatric surveillance	Excluded	No relevent information
R. Hoque, S. M. Ahmed, N. Naher, M. A. Islam, E. K. Rousham, B. Z. Islam and S. Hassan	2020	Tackling antimicrobial resistance in Bangladesh: A scoping review of policy and practice in human, animal and environment sectors	Excluded	Review paper
R. Hoque, A. Mostafa and M. Haque	2015	Intern doctors’ views on the current and future antibiotic resistance situation of Chattagram Maa O Shishu Hospital Medical College, Bangladesh	Excluded	No relevent information
A. Hossain, S. Nakamichi, M. Habibullah-Al-Mamun, K. Tani, S. Masunaga and H. Matsuda	2017	Occurrence, distribution, ecological and resistance risks of antibiotics in surface water of finfish and shellfish aquaculture in Bangladesh	Excluded	No relevent information
M. Hossain, S. Biswas and Z. Marufee	2016	Study of some commonly prescribed antibiotics against some infectious diseases in Bangladesh	Excluded	Review paper
M. J. Hossain, M. S. Islam, M. A. Sobur, S. B. Zaman, A. Nahar, M. Rahman and M. T. Rahman	2020	Exploring poultry farm environment for antibiotic resistant Escherichia coli, Salmonella spp., and Staphylococcus spp. having public health significance	Excluded	No relevent information
U. A. Imam, A. Zahrau and E. I. Obeagu		The threat of Antimicrobial Resistance in Developing Countries: Causes and Control Strategies	Excluded	Not country specific
S. Imanpour, O. Nwaiwu, D. K. McMaughan, B. DeSalvo and A. Bashir	2017	Factors associated with antibiotic prescriptions for the viral origin diseases in office-based practices, 2006–2012	Excluded	Not country specific
H. Iqbal, N. Mahboob, M. Ahmed, K. Z. Mamun, A. Rahim and K. A. K. Azad	2018	Bacteriological profile and antimicrobial susceptibility patterns of blood culture isolates among febrile patients in a Tertiary care hospital, Bangladesh	Excluded	No gender segregated information
K. Iskandar, L. Molinier, S. Hallit, M. Sartelli, F. Catena, F. Coccolini, T. Craig Hardcastle, C. Roques and P. Salameh	2020	Drivers of antibiotic resistance transmission in low-and middle-income countries from a “one health” perspective—a review	Excluded	Review paper
K. Iskandar, L. Molinier, S. Hallit, M. Sartelli, T. C. Hardcastle, M. Haque, H. Lugova, S. Dhingra, P. Sharma and S. Islam	2021	Surveillance of antimicrobial resistance in low-and middle-income countries: a scattered picture	Excluded	No gender segregated information
B. Islam, M. K. Paul, A. A. Sweety, M. Farhad, F. Ahmed, L. N. Nizhu, K. Begum, I. A. Chowdhury and H. H. Rahman	2018	Current trends in prescribing antimicrobial drugs on patients suffering from ENT infection in a teaching hospital in Khulna	Excluded	No gender segregated information
M. A. Islam, S. Begum, S. S. Parul, A. Bhuyian, M. T. Islam and M. K. Islam	2019	Antibiotic resistance pattern in children with UTI: A study in a Tertiary Care Hospital, Dhaka, Bangladesh	Excluded	No gender segregated information
M. M. Islam	2021	Bacterial resistance to antibiotics: access, excess, and awareness in Bangladesh	Excluded	No relevent information
M. S. Islam, K. M. Y. K. Sikdar, A. S. M. M. A. L. Hossain and A. B. M. Faroque	2019	Study on the pattern of antibiotic use including the resistance episodes in Bangladesh	Excluded	No gender segregated information
M. T. Islam	2023	Bacteriological Profile and Antimicrobial Resistance Patterns in Pediatric Urinary Tract Infections: A Cross-Sectional Study at Dhaka Shishu Hospital	Excluded	Full text not available
M. Z. Islam, M. S. Islam, L. R. Kundu, A. Ahmed, K. Hsan, S. Pardhan, R. Driscoll, M. S. Hossain and M. M. Hossain	2022	Knowledge, attitudes and practices regarding antimicrobial usage, spread and resistance emergence in commercial poultry farms of Rajshahi district in Bangladesh	Excluded	No gender segregated information
Y. Jahan, K. Begum, M. Murshed, A. Kabir and K. Begum	1999	Multiple drug resistant Neisseria gonorrhoeae among females of high risk behaviour in Dhaka city	Excluded	Full text not available
K. S. Kabir	2023	Experience of the hijra community in accessing formal healthcare in Dhaka city: an exploratory qualitative study	Excluded	No AMR related information
A. Kalam, S. Shano, M. A. Khan, A. Islam, N. Warren, M. M. Hassan and M. Davis	2021	Understanding the social drivers of antibiotic use during COVID-19 in Bangladesh: Implications for reduction of antimicrobial resistance	Excluded	No gender segregated information
M. A. Kalam, M. A. Alim, S. Shano, M. R. K. Nayem, M. R. Badsha, M. A. Al Mamun, A. Hoque, A. Z. Tanzin, S. Ali Khan, A. Islam, M. M. Islam and M. M. Hassan	2021	Knowledge, attitude, and practices on antimicrobial use and antimicrobial resistance among poultry drug and feed sellers in Bangladesh	Excluded	No gender segregated information
M. A. Kawsar, M. T. Alam, D. Pandit, M. M. Rahman, M. Mia, A. Talukdar and T. A. Sumon	2022	Status of disease prevalence, drugs and antibiotics usage in pond-based aquaculture at Narsingdi district, Bangladesh: A major public health concern and strategic appraisal for mitigation	Excluded	Wrong outcome
S. Khan, S. S. Hasan, S. E. Bond, B. R. Conway and M. A. Aldeyab	2022	Antimicrobial consumption in patients with COVID-19: a systematic review and meta-analysis	Excluded	Review paper
F. Khanam, M. A. Sayeed, F. K. Choudhury, A. Sheikh, D. Ahmed, D. Goswami, M. L. Hossain, A. Brooks, S. B. Calderwood, R. C. Charles, A. Cravioto, E. T. Ryan and F. Qadri	2015	Typhoid fever in young children in Bangladesh: clinical findings, antibiotic susceptibility pattern and immune responses	Excluded	No gender segregated information
H. Khatun, S. B. Islam, N. N. Naila, S. A. Islam, B. Nahar, N. H. Alam and T. Ahmed	2018	Clinical profile, antibiotic susceptibility pattern of bacterial isolates and factors associated with complications in culture-proven typhoid patients admitted to an urban hospital in Bangladesh	Excluded	No gender segregated information
R. King, J. Hicks, C. Rassi, M. Shafique, D. Barua, P. Bhowmik, M. Das, H. Elsey, K. Questa, F. Fieroze, P. Hamade, S. Huque, J. Newell and R. Huque	2020	A process for developing a sustainable and scalable approach to community engagement: community dialogue approach for addressing the drivers of antibiotic resistance in Bangladesh	Excluded	No gender segregated information
P. J. Lucas, M. R. Uddin, N. Khisa, S. M. S. Akter, L. Unicomb, P. Nahar, M. A. Islam, F. A. Nizame and E. K. Rousham	2019	Pathways to antibiotics in Bangladesh: A qualitative study investigating how and when households access medicine including antibiotics for humans or animals when they are ill	Excluded	No gender segregated information
S. Mah-E-muneer, M. Z. Hassan, M. A. A. J. Biswas, F. Rahman, Z. Akhtar, P. Das, M. A. Islam and F. Chowdhury	2021	Use of antimicrobials among suspected covid-19 patients at selected hospitals, bangladesh: Findings from the first wave of covid-19 pandemic	Excluded	No gender segregated information
A. Masud, R. L. Walpola, M. Sarker, A. Kabir, M. Asaduzzaman, M. S. Islam, A. T. Mostafa, Z. Akhtar, M. Barua and H. Seale	2024	Understanding Antibiotic Purchasing Practices in Community Pharmacies in Bangladesh: A Potential Driver of Emerging Antimicrobial Resistance	Excluded	Not peer reviewed
M. A. Matin, W. A. Khan, M. M. Karim, S. Ahmed, J. John-Langba, O. A. Sankoh, M. Gyapong, J. Kinsman and H. Wertheim	2020	What influences antibiotic sales in rural Bangladesh? A drug dispensers’ perspective	Excluded	No gender segregated information
R. S. Md Rezal, M. A. Hassali, A. A. Alrasheedy, F. Saleem, F. A. Md Yusof and B. Godman	2015	Physicians’ knowledge, perceptions and behaviour towards antibiotic prescribing: a systematic review of the literature	Excluded	Review paper
M. F. Mia, A. A. Mia, D. Saha, A. A. Khan and A. Chakraborty	2017	Antibiotic resistance pattern and stewardship programme in critical care settings	Excluded	No gender segregated information
H. Momtaz, N. Tomalika, M. Mohsena, M. M. Rhahman, N. Sultana and M. A. Sayeed	2018	Morbidity and drug prescribing patterns at a rural primary health care center of Bangladesh	Excluded	No gender segregated information
U. K. Mondal, T. Haque, M. A. A. J. Biswas, S. M. Satter, M. S. Islam, Z. Alam, M. Shojon, S. Debnath, M. Islam and H. B. Murshid	2022	Antibiotic Prescribing practices for treating COVID-19 patients in Bangladesh	Excluded	No gender segregated information
M. S. Moonajilin, M. A. Mamun, M. E. Rahman, M. F. Mahmud, A. H. M. S. Al Mamun, M. S. Rana and D. Gozal	2020	Prevalence and drivers of self-medication practices among savar residents in Bangladesh: A cross-sectional study	Excluded	No gender segregated information
B. W. Morgan, M. R. Grigsby, T. Siddharthan, M. Chowdhury, A. Rubinstein, L. Gutierrez, V. Irazola, J. J. Miranda, A. Bernabe-Ortiz, D. Alam, R. A. Wise and W. Checkley	2019	Epidemiology and risk factors of asthma-chronic obstructive pulmonary disease overlap in low- and middle-income countries	Excluded	No gender segregated information
S. M. Muflih, S. Al-Azzam, R. A. Karasneh, B. R. Conway and M. A. Aldeyab	2021	Public health literacy, knowledge, and awareness regarding antibiotic use and antimicrobial resistance during the COVID-19 pandemic: A cross-sectional study	Excluded	Not country specific
J. L. Murray, D. T. Leung, O. R. Hanson, S. M. Ahmed, A. T. Pavia, A. I. Khan, J. E. Szymczak, V. M. Vaughn, P. K. Patel and D. Biswas	2024	Drivers of inappropriate use of antimicrobials in South Asia: A systematic review of qualitative literature	Excluded	Review paper
P. Nahar, L. Unicomb, P. J. Lucas, M. R. Uddin, M. A. Islam, F. A. Nizame, N. Khisa, S. M. S. Akter and E. K. Rousham	2020	Antibiotic Prescribing practices for treating COVID-19 patients in Bangladesh	Excluded	Wrong outcome
M. Nasir, A. Chowdhury and T. Zahan	2020	Self-medication during COVID-19 outbreak: a cross sectional online survey in Dhaka city	Excluded	No gender segregated information
M. Nasir, K. A. Talha, A. S. M. S. Chowdhury, T. Zahan and R. A. Perveen	2020	Prevalence, pattern and impact of self medication of anti-infective agents during COVID-19 outbreak in Dhaka City	Excluded	No gender segregated information
S. Nazrina, M. S. Rahman and R. Naznin	2020	Antibiotic Dispensing Practice by Medicine Sellers in Dhaka City-A Cross-Sectional Study	Excluded	Wrong outcome
F. Noman, G. A. Zahan, F. Hasnat and M. K. Islam	2020	Sensitivity and resistant pattern of commonly used drugs in uti in younger children: A Study in a Tertiary Care Level Hospital, Dhaka, Bangladesh	Excluded	No gender segregated information
T. Nusrat, N. Akter, N. A. A. Rahman, B. Godman, D. T. D. Rozario and M. Haque	2020	Antibiotic resistance and sensitivity pattern of Metallo-β-Lactamase Producing Gram-Negative Bacilli in ventilator-associated pneumonia in the intensive care unit of a public medical school hospital in Bangladesh	Excluded	No gender segregated information
S. Nuzhat, R. Das, S. Das, S. B. Islam, P. Palit, M. A. Haque, S. Chakraborty, S. H. Khan, D. Ahmed, B. Alam, T. Ahmed, M. J. Chisti and A. S. G. Faruque	2022	Antimicrobial resistance in shigellosis: A surveillance study among urban and rural children over 20 years in Bangladesh	Excluded	No gender segregated information
M. A. G. Rabbani, M. Z. H. Howlader and Y. Kabir	2017	Detection of multidrug resistant (MDR) bacteria in untreated waste water disposals of hospitals in Dhaka City, Bangladesh	Excluded	No gender segregated information
M. A. Rafi, M. M. Z. Miah, M. A. Wadood and M. G. Hossain	2020	Risk factors and etiology of neonatal sepsis after hospital delivery: A case-control study in a tertiary care hospital of Rajshahi, Bangladesh	Excluded	No gender segregated information
M. Rahman and S. Islam	2019	Prescribing trends in the outpatient department in a rural hospital in Bangladesh	Excluded	No gender segregated information
M. A. Rahman, A. Rahman, M. A. Islam and M. M. Alam	2017	Antimicrobial resistance of Escherichia coli isolated from milk, beef and chicken meat in Bangladesh	Excluded	Wrong outcome
M. H. Rahman, S. Agarwal, S. Tuddenham, H. Peto, M. Iqbal, A. Bhuiya and D. H. Peters	2015	What do they do? Interactions between village doctors and medical representatives in Chakaria, Bangladesh	Excluded	Wrong outcome
M. M. Rahman, M. A. Alam Tumpa, M. Zehravi, M. T. Sarker, M. D. Yamin, M. R. Islam, M. Harun-Or-Rashid, M. Ahmed, S. Ramproshad and B. Mondal	2022	An overview of antimicrobial stewardship optimization: the use of antibiotics in humans and animals to prevent resistance	Excluded	Review paper
T. Rahman, M. S. Islam, S. Paul, M. M. Islam, M. A. Samadd, R. N. Reyda and M. R. Sarkar	2023	Prescription patterns in an intensive care unit of COVID-19 patients in Bangladesh: A cross-sectional study	Excluded	No gender segregated information
M. H. Rashid, M. R. Md, S. Hafiza, K. I. Md, A. K. Muhammad and S. E. Syeda	2020	Antibacterial resistance patterns of bacteria isolated from clinical specimens at Uttara IbnSina Diagnostic Centre, Dhaka	Excluded	No gender segregated information
M. M. Rashid, Z. Akhtar, S. Chowdhury, M. A. Islam, S. Parveen, P. K. Ghosh, A. Rahman, Z. H. Khan, K. Islam, N. Debnath, M. Rahman and F. Chowdhury	2022	Pattern of Antibiotic Use among Hospitalized Patients according to WHO Access, Watch, Reserve (AWaRe) Classification: Findings from a Point Prevalence Survey in Bangladesh	Excluded	No gender segregated information
R. S. Rasu, M. Iqbal, S. M. A. Hanifi, A. Moula, S. Hoque, S. Rasheed and A. Bhuiya	2014	Level, pattern, and determinants of polypharmacy and inappropriate use of medications by village doctors in a rural area of Bangladesh	Excluded	No gender segregated information
M. Rifat, J. Hall, C. Oldmeadow, A. Husain and A. H. Milton	2015	Health system delay in treatment of multidrug resistant tuberculosis patients in Bangladesh	Excluded	No gender segregated information
S. G. Rizvi and S. Z. Ahammad	2022	COVID-19 and antimicrobial resistance: A cross-study	Excluded	Review paper
F. Rizwan, F. Monjur, M. Asaduzzaman, N. Nasrin, N. Ghosh, A. K. M. Samsuzzaman, S. A. Afzal, A. S. Apu, M. Amran Howlader and A. F. M. Salim	2012	Antibiotic susceptibility patterns of uropathogens isolated from pediatric patients in a selected hospital of Bangladesh	Excluded	Timeframe
A. A. Roess, P. J. Winch, N. A. Ali, A. Akhter, D. Afroz, S. El Arifeen, G. L. Darmstadt and A. H. Baqui	2013	Animal husbandry practices in rural Bangladesh: Potential risk factors for antimicrobial drug resistance and emerging diseases	Excluded	Timeframe
E. K. Rousham, M. A. Islam, P. Nahar, P. J. Lucas, N. Naher, S. M. Ahmed, F. A. Nizame and L. Unicomb	2019	Pathways of antibiotic use in Bangladesh: qualitative protocol for the PAUSE study	Excluded	Protocol
S. Saha and M. T. Hossain	2017	Evaluation of medicines dispensing pattern of private pharmacies in Rajshahi, Bangladesh	Excluded	No gender segregated information
S. K. Saha and S. Promite	2017	Factors influencing clinician’s antibiotic prescribing behaviors (APB) in Bangladesh: An in-depth review using COMB Model	Excluded	Review paper
M. S. I. Sajib, A. M. Tanmoy, Y. Hooda, H. Rahman, S. J. Munira, A. Sarkar, D. Das, M. A. Rahman, N. Islam, M. Shahidullah, M. R. Amin, M. J. Alam, M. Hanif, S. P. Luby, D. O. Garrett, S. K. Saha and S. Sahaid	2023	Trends in antimicrobial resistance amongst Salmonella Paratyphi A isolates in Bangladesh: 1999–2021	Excluded	No gender segregated information
N. Samir, M. Z. Hassan, M. Biswas, F. Chowdhury, Z. Akhtar, R. Lingam, S. Banu and N. Homaira	2021	Antibiotic Use for Febrile Illness among Under-5 Children in Bangladesh: A Nationally Representative Sample Survey	Excluded	No gender segregated information
A. A. Sani, K. Rafiq, F. Akter, P. Islam, S. Sachi, N. Sultana, S. Hayat, U. B. Usman, M. S. Islam and M. Z. Islam	2023	Effect of knowledge of informal poultry drug prescribers on their attitude and practice toward antimicrobial use, residues, and resistance in Bangladesh	Excluded	No gender segregated information
S. Sattar, R. Quddus and S. K. Saha	2018	Pattern of Self-Medication Practices among Rural Population of Mymensingh	Excluded	Full text not available
M. A. Sayeed, N. Iqbal, M. S. Ali, M. M. Rahman, M. R. Islam and M. Jakaria	2015	Survey on antibiotic practices in chittagong city of Bangladesh	Excluded	No gender segregated information
N. A. Siddiky, S. Islam, M. S. Sarker, R. Begum and M. A. Samad	2022	Knowledge, Attitude and Practices of Poultry Farmers on Antimicrobial Use, Resistance and Farm Hygiene Management in Bangladesh	Excluded	Not peer reviewed
M. J. Sujan, Z. H. Habib, A. Rahman, S. M. S. Rizvi, H. T. Barua, A. T. Aboushady, M. A. Hasnat, S. B. G. Rasul, H. S. Joh, K. Prifti, K. K. Chi, S. Y. Kwon, A. Clark, S. Gautam, M. Holm, F. Marks, J. Stelling, A. Shaw and N. Poudyal	2023	Antimicrobial Resistance Surveillance Methods in Bangladesh: Present and Way Forward	Excluded	No gender segregated information
S. P. Sultana and M. S. Rahman	2017	Dynamic online antimicrobial guideline with stewardship program: Impact on antimicrobial prescribing	Excluded	No gender segregated information
S. A. Sumon, M. M. U. Anwar, F. M. Akther, A. S. Priyanka, T. Tamanna, A. Rahman, M. S. Islam and M. G. D. Harun	2024	Perceptions of antibiotic stewardship programmes and determinants of antibiotic prescribing patterns among physicians in tertiary hospitals in Bangladesh: implications for future policy and practice	Excluded	No gender segregated information
A. M. Tanmoy, E. Westeel, K. De Bruyne, J. Goris, A. Rajoharison, M. S. I. Sajib, A. van Belkum, S. K. Saha, F. Komurian-Pradel and H. P. Endtz	2018	Salmonella enterica Serovar Typhi in Bangladesh: Exploration of Genomic Diversity and Antimicrobial Resistance	Excluded	No gender segregated information
M. M. Tohan, F. Ahmed, I. J. Juie, A. Kabir, M. H. Howlader and M. A. Rahman	2024	Knowledge attitude and convenience on self-medication practices among university students in Bangladesh exploration using structural equation modeling approach	Excluded	No gender segregated information
L. E. Unicomb, F. A. Nizame, M. R. Uddin, P. Nahar, P. J. Lucas, N. Khisa, S. M. S. Akter, M. A. Islam, M. Rahman and E. K. Rousham	2021	Motivating antibiotic stewardship in Bangladesh: identifying audiences and target behaviours using the behaviour change wheel	Excluded	No gender segregated information
H. F. L. Wertheim, N. T. K. Chuc, S. Punpuing, W. A. Khan, M. Gyapong, K. P. Asante, K. Munguambe, F. X. Gómez-Olivé, P. Ariana and J. John-Langba	2017	Community-level antibiotic access and use (ABACUS) in low-and middle-income countries: Finding targets for social interventions to improve appropriate antimicrobial use–an observational multi-centre study	Excluded	Not country specific

## Sheet: Sri Lanka

Author name	Year	Title	Decision	Reason for exclusion
N. Amarasinghe, M. Athavan, D. Jayamanne, Y. Rajapakshe, A. Sadikeen, K. Gunasekara, A. Fernando and L. Karunanayake	2018	Bacterial profile and antibiotic susceptibility pattern of adult lower respiratory tract infections in Colombo, Sri Lanka	Excluded	No gender segregated information
S. Ariyawansa, K. N. Gunawardana, M. M. Hapudeniya, N. J. Manelgamage, C. R. Karunarathne, R. P. Madalagama, K. H. Ubeyratne, D. Wickramasinghe, H. M. Tun and P. Wu	2023	One Health surveillance of antimicrobial use and resistance: challenges and successes of implementing surveillance programs in Sri Lanka	Excluded	No gender segregated information
H. E. F. Carvalho, G. Schneider, A. G. dos Santos Junior, O. M. A. Batista, J. Mondlane, A. M. Ferreira, D. de Andrade and A. R. M. da Costa Valle	2021	Prescription of antimicrobials in primary health care: scoping review	Excluded	Review paper
E. Charani, M. Mendelson, S. J. Pallett, R. Ahmad, M. Mpundu, O. Mbamalu, C. Bonaconsa, V. Nampoothiri, S. Singh and N. Peiffer-Smadja	2023	An analysis of existing national action plans for antimicrobial resistance—gaps and opportunities in strategies optimising antibiotic use in human populations	Excluded	Review paper
G. Chathuranga, T. Dissanayake, N. Fernando and C. Wanigatunge	2021	Appropriateness of the empirical antibiotics prescribed and their concordance with national guidelines for three selected infections among cancer patients in a tertiary care centre in Sri Lanka	Excluded	No gender segregated information
T. Cooray, J. Zhang, H. Zhong, L. Zheng, Y. Wei, S. K. Weragoda, K. Jinadasa and R. Weerasooriya	2021	Profiles of antibiotic resistome and microbial community in groundwater of CKDu prevalence zones in Sri Lanka	Excluded	No gender segregated information
R. Dacombe, I. Bates, M. Bhardwaj, S. Wallis and J. Pulford	2016	Fleming Fund: supporting surveillance capacity for antimicrobial resistance An analysis of approaches to laboratory capacity strengthening for drug resistant infections in low and middle income countries	Excluded	Report
H. Daraniyagala, O. Dahanayake, A. Dasanayake, P. Dayarathna, S. Dayarathna, K. Dayasiri, D. De Silva, S. De Silva, N. De Silva and D. De Silva	2023	Contamination of Clinical White Coats with Potential Pathogens and their Antibiotic Resistant Phenotypes Among a Group of Sri Lankan Medical Students	Excluded	No gender segregated information
B. Devakanthan, M. Kothalawala, K. Galgamuwa, D. Yatigammana, G. L. Kumarage, P. Paththamperuma, S. Arunan and V. Liyanapathirana	2021	Point prevalence survey of antimicrobial prescriptions in selected units of National Hospital Kandy, Sri Lanka	Excluded	No gender segregated information
Dolamullla	2021	Reconsidering middle-Income country approaches to a global antimicrobial resistance (AMR) problem: A case study of Sri Lanka	Excluded	Not peer reviewed
A. M. Dondorp, D. Limmathurotsakul and E. A. Ashley	2018	What’s wrong in the control of antimicrobial resistance in critically ill patients from low-and middle-income countries?	Excluded	Review paper
L. Duan, C. Liu, D. Wang, R. Lin, P. Qian, X. Zhang and C. Liu	2022	The vicious cycle of the public's irrational use of antibiotics for upper respiratory tract infections: A mixed methods systematic review	Excluded	Review paper
T. A. Esteves, G. J. Atabay and E. Y. Tan	2023	Determinants of dispensing antibiotics without prescription by community pharmacists in developing countries: A review	Excluded	Review paper
A. D. A. Fernando, L. M. H. Bandara, H. Bandara, S. Pilapitiya and A. de Silva	2017	A descriptive study of self-medication practices among Sri Lankan national level athletes	Excluded	No gender segregated information
B. Fernandopulle, K. Yasarathna, A. Gunasekara, K. Wijesinghe, B. Beneragama, L. Panapitiya, L. Wanniarachchi and S. S. Ranganathan	2020	Antibiotic Consumption in the State Sector of Sri Lanka Over 25 Years: 1994-2018 Using the GAP Methodology	Excluded	No gender segregated information
P. Galappatthy, P. Ranasinghe, C. K. Liyanage, M. Wijayabandara, D. S. Warapitiya, D. Jayasekara and R. L. Jayakody	2021	Core prescribing indicators and the most commonly prescribed medicines in a tertiary health care setting in a developing country	Excluded	No gender segregated information
S. Gandra, G. Alvarez-Uria, P. Turner, J. Joshi, D. Limmathurotsakul and H. R. van Doorn	2020	Antimicrobial resistance surveillance in low-and middle-income countries: progress and challenges in eight South Asian and Southeast Asian countries	Excluded	Review paper
Y. D. Gunasekara, T. Kinnison, S. A. Kottawatta, R. S. Kalupahana and A. Silva-Fletcher	2022	Exploring barriers to one health antimicrobial stewardship in Sri Lanka: a qualitative study among healthcare professionals	Excluded	No gender segregated information
K. S. Guruge, Y. A. Tamamura, P. Goswami, R. Tanoue, K. Jinadasa, K. Nomiyama, T. Ohura, T. Kunisue, S. Tanabe and M. Akiba	2021	The association between antimicrobials and the antimicrobial-resistant phenotypes and resistance genes of Escherichia coli isolated from hospital wastewaters and adjacent surface waters in Sri Lanka	Excluded	No gender segregated information
M. Haque	2017	Antimicrobial use, prescribing, and resistance in selected ten selected developing countries: a brief overview	Excluded	Review paper
K. Hathagoda, S. Gunathilaka, B. Dissanayake, I. Tharaka, K. Gunathilaka, R. Jayawardane and R. Gunapala	2018	Bacterial pathogens causing urinary tract infections in children and their antimicrobial susceptibility patterns in a tertiary care hospital in Sri Lanka	Excluded	No gender segregated information
E. Herath, A. Palansooriya, W. Dandeniya and R. Jinadasa	2016	An assessment of antibiotic resistant bacteria in poultry litter and agricultural soils in Kandy district, Sri Lanka	Excluded	No gender segregated information
M. S. Hossain, M. F. Islam, P. B. Arka, M. Rohman, T. S. Ahmed, T. Ahammed, M. A. B. Chowdhury and M. J. Uddin	2023	Antibiotic prescription from qualified sources for children with fever/cough: cross-sectional study from 59 low-and middle-income countries	Excluded	Not country specific
S. C. Illapperuma, S. B. Agampodi and E. M. Corea	2019	Bacterial aetiology and antimicrobial resistance pattern in culture positive enteric fever at a private hospital in Sri Lanka	Excluded	No gender segregated information
V. Ivanovska, B. Angelovska, L. van Dijk, M. Zdravkovska, H. G. Leufkens and A. K. Mantel-Teeuwisse	2017	CHANGE IN PARENTAL KNOWLEDGE, ATTITUDES AND PRACTICE OF ANTIBIOTIC USE AFTER IMPLEMENTATION OF A NATIONAL INTERVENTION PROGRAMME	Excluded	No gender segregated information
K. Jayatilleke	2014	Increasing antibiotic resistance in a tertiary care hospital in Sri Lanka	Excluded	No gender segregated information
J. Jayaweera and M. Reyes	2018	Antimicrobial misuse in pediatric urinary tract infections: recurrences and renal scarring	Excluded	No gender segregated information
T. Jayaweera, H. Ruwandeepika, V. Deekshit, J. Vidanarachchi, S. Kodithuwakku, I. Karunasagar and H. Cyril	2020	Isolation and Identification of Salmonella spp. from Broiler Chicken Meat in Sri Lanka and their Antibiotic Resistance	Excluded	Wrong outcome
M. Jayaweerasingham, S. Angulmaduwa and V. Liyanapathirana	2019	Knowledge, beliefs and practices on antibiotic use and resistance among a group of trainee nurses in Sri Lanka	Excluded	No gender segregated information
M. Jones and C. Liyanage	2018	Traditional medicine and primary health care in Sri Lanka: Policy, perceptions, and practice	Excluded	Not AMR related
I. Karunarathna, A. Jayawardana, S. Bandara and M. Abeykoon		Optimizing Antibiotic Use: Strategies and Challenges in Antimicrobial Stewardship Programs	Excluded	No gender segregated information
M. Kumar, G. T. Chaminda and R. Honda	2020	Seasonality impels the antibiotic resistance in Kelani River of the emerging economy of Sri Lanka	Excluded	Wrong outcome
M. Kumar, T. Chaminda, A. K. Patel, H. Sewwandi, P. Mazumder, M. Joshi and R. Honda	2020	Prevalence of antibiotic resistance in the tropical rivers of Sri Lanka and India	Excluded	Wrong outcome
M. Kumar, B. Ram, H. Sewwandi, R. Honda and T. Chaminda	2020	Treatment enhances the prevalence of antibiotic-resistant bacteria and antibiotic resistance genes in the wastewater of Sri Lanka, and India	Excluded	Wrong outcome
G. Y. Liyanage and P. M. Manage	2016	Occurrence, fate and ecological risk of antibiotics in hospital effluent water and sediments in Sri Lanka	Excluded	No gender segregated information
P. G. Medrano, N. Weerasinghe, A. Nagahawatte, S. Vanderburg, L. P. Park, G. B. Wijayaratne, V. Devasiri, B. Dilshan, T. Sheng, R. Kurukulasooriya, J. Anderson, B. P. Nicholson, C. W. Woods, C. K. Bodinayake and L. G. Tillekeratne	2024	Prevalence and predictors of antibiotic prescription among patients hospitalized with viral lower respiratory tract infections in Southern Province, Sri Lanka	Excluded	No gender segregated information
R. Morimoto	2023	Comparing awareness of antibiotic resistance in Sri Lanka and the European Union	Excluded	No gender segregated information
J. L. Murray, D. T. Leung, O. R. Hanson, S. M. Ahmed, A. T. Pavia, A. I. Khan, J. E. Szymczak, V. M. Vaughn, P. K. Patel and D. Biswas	2024	Drivers of inappropriate use of antimicrobials in South Asia: A systematic review of qualitative literature	Excluded	Review paper
W. H. Organization	2018	WHO country cooperation strategy: 2018–2023, Sri Lanka	Excluded	Report
W. H. Organization	2018	WHO Sri Lanka annual report, 2017	Excluded	Report
W. H. Organization	2018	WHO report on surveillance of antibiotic consumption: 2016-2018 early implementation	Excluded	Report
W. H. Organization	2019	WHO Sri Lanka Biennium Report 2018-2019	Excluded	Report
K. Perera, B. Chamara, W. Amarasinghe, M. Ariyadasa, H. Masimbula, H. De Silva and S. Kumari	2022	Evaluation of Practice on Self-Medication among Allied Health Science Undergraduates of Sri Lanka, Therapeutic and Toxicity Implications	Excluded	No gender segregated information
A.-C. Peters, D. J. Larsson, R. Laxminarayan and C. Munthe	2024	Barriers and pathways to environmental surveillance of antibiotic resistance in middle-and low-income settings: a qualitative exploratory key expert study	Excluded	Not country specific
D. Rathish, S. Bahini, T. Sivakumar, T. Thiranagama, T. Abarajithan, B. Wijerathne, C. Jayasumana and S. Siribaddana	2016	Drug utilization, prescription errors and potential drug-drug interactions: an experience in rural Sri Lanka	Excluded	No gender segregated information
M. Sakeena, A. Bennett and A. McLachlan	2020	Strategies to be Implemented in Sri Lanka to Improve Appropriate Antibiotic Dispensing Practices: A Narrative Review	Excluded	Review paper
M. Sakeena, A. A. Bennett, S. Jamshed, F. Mohamed, D. R. Herath, I. Gawarammana and A. J. McLachlan	2018	Investigating knowledge regarding antibiotics and antimicrobial resistance among pharmacy students in Sri Lankan universities	Excluded	No gender segregated information
M. H. Sakeena, A. A. Bennett, F. Mohamed, H. M. Herath, I. Gawarammane and A. J. McLachlan	2018	Investigating knowledge regarding antibiotics among pharmacy and allied health sciences students in a Sri Lankan university	Excluded	No gender segregated information
M. H. F. Sakeena, A. A. Bennett, S. J. Carter and A. J. McLachlan	2019	A comparative study regarding antibiotic consumption and knowledge of antimicrobial resistance among pharmacy students in Australia and Sri Lanka	Excluded	Not country specific
G. G. Senadheera, S. S. Ranganathan, G. Fernando and B. Fernandopulle	2019	Antibacterial medicine consumption in private and state sector outpatient settings in colombo district, Sri Lanka	Excluded	No gender segregated information
G. P. Senadheera, S. Sri Ranganathan, G. Patabendige, G. H. Fernando, D. Gamage, R. M. Maneke and B. M. Fernandopulle	2016	Resistance and utilisation pattern of antibacterial agents in outpatient settings in two Teaching Hospitals in Colombo	Excluded	No gender segregated information
T. Sheng, G. B. Wijayaratne, T. M. Dabrera, R. J. Drew, A. Nagahawatte, C. K. Bodinayake, R. Kurukulasooriya, T. Østbye, K. J. Nagaro, C. De Silva, H. Ranawakaarachchi, A. T. Sudarshana, D. J. Anderson, C. W. Woods and L. G. Tillekeratne	2019	Point-prevalence study of antimicrobial use in public hospitals in southern Sri Lanka identifies opportunities for improving prescribing practices	Excluded	No gender segregated information
T. Sheng, G. B. Wijayaratne, T. M. Dabrera, A. Nagahawatte, C. K. Bodinayake, R. Kurukulasooriya, et al.	2019	Skin and Soft-tissue Infections Are a Common Reason for Potentially Inappropriate Antimicrobial Use among Inpatients in Sri Lanka	Excluded	No gender segregated information
B. Sihombing, R. Bhatia, R. Srivastava, T. Y. Aditama, R. Laxminarayan and S. Rijal	2023	Response to antimicrobial resistance in South-East Asia region	Excluded	Not country specific
C. Tarrant, A. M. Colman, D. R. Jenkins, E. Chattoe-Brown, N. Perera, S. Mehtar, W. D. Nakkawita, M. Bolscher and E. M. Krockow	2021	Drivers of broad-spectrum antibiotic overuse across diverse hospital contexts—A qualitative study of prescribers in the UK, Sri Lanka and South Africa	Excluded	No gender segregated information
C. Tarrant, E. M. Krockow, W. D. Nakkawita, M. Bolscher, A. M. Colman, E. Chattoe-Brown, N. Perera, S. Mehtar and D. R. Jenkins	2020	Moral and contextual dimensions of “inappropriate” antibiotic prescribing in secondary care: a three-country interview study	Excluded	No gender segregated information
L. G. Tillekeratne	2014	Impact of a Point-of-Care Rapid Influenza Test on Antibiotic Prescribing Patterns in Southern Sri Lanka	Excluded	Not peer reviewed
L. G. Tillekeratne, C. K. Bodinayake, T. Dabrera, A. Nagahawatte, W. K. Arachchi, A. Sooriyaarachchi, K. Stewart, M. Watt, T. Østbye and C. W. Woods	2017	Antibiotic overuse for acute respiratory tract infections in Sri Lanka: a qualitative study of outpatients and their physicians	Excluded	No gender segregated information
A. Tompson, L. Manderson and C. Chandler	2021	Understanding antibiotic use: practices, structures and networks	Excluded	No gender segregated information
A. C. Tompson and C. I. Chandler	2021	Addressing antibiotic use: insights from social science around the world	Excluded	Not country specific
N. Torres, B. Chibi, L. Middleton, V. Solomon and T. Mashamba-Thompson	2019	Evidence of factors influencing self-medication with antibiotics in low and middle-income countries: a systematic scoping review	Excluded	Review paper
N. F. Torres, B. Chibi, D. Kuupiel, V. P. Solomon, T. P. Mashamba-Thompson and L. E. Middleton	2021	The use of non-prescribed antibiotics; prevalence estimates in low-and-middle-income countries. A systematic review and meta-analysis	Excluded	Review paper
D. T. van Melle, G. Ten Asbroek, R. Rolfe, S. Vanderburg, Y. W. Abeysinghe, C. Halloluwa, H. L. Zhang, T. Ostbye, R. Kurukulasooriya, T. Sheng, S. Kanchana, G. Wijayaratne, C. Bodinayake, A. Nagahawatte, M. H. Watt, C. W. Woods, V. de Silva and G. Tillekeratne	2021	Knowledge, Perceptions, and Attitudes Regarding Antibiotic Use for Lower Respiratory Tract Infections: Insights from Patients in Sri Lanka	Excluded	No gender segregated information
A. Wilkinson, A. Ebata and H. MacGregor	2018	Interventions to reduce antibiotic prescribing in LMICs: a scoping review of evidence from human and animal health systems	Excluded	Review paper
A. Willemsen, S. Reid and Y. Assefa	2022	A review of national action plans on antimicrobial resistance: strengths and weaknesses	Excluded	Review paper
M. S. Zawahir	2019	Pharmacists’ provision and public’s use of antibiotics for common infections in Sri Lanka	Excluded	Not peer reviewed
S. Zawahir, S. Lekamwasam and P. Aslani	2021	Factors related to antibiotic supply without a prescription for common infections: a cross-sectional national survey in Sri Lanka	Excluded	No gender segregated information
Giri, S., Ghimire, A., Mishra, A., Acharya, K., Kuikel, S., Tiwari, A. and Mishra, S.K., 2023. Prevalence of methicillin-resistant Staphylococcus aureus carriage among healthcare workers in South Asia in non-outbreak settings: A systematic review and meta-analysis. American Journal of Infection Control, 51(2), pp.184-193			Excluded	Review paper
