# Supplementary material for: Gender and equity within antimicrobial resistance: a scoping review of evidence and policy in Bangladesh and Sri Lanka
Source: BMC Public Health. 2026 Jun 4;26:2313. doi: 10.1186/s12889-026-27842-y (PMC13455460; doi:10.1186/s12889-026-27842-y)
Supplement: Supplementary file 2 [file 12889_2026_27842_MOESM11_ESM.docx]

**Additional File 11: Framework for analysis**

| **Theme** | **Sub-theme** |
| --- | --- |
| Vulnerability of infection | - Clinical susceptibility   - Biological and anatomical factors influencing infection risks. - Exposure to infection   - Occupational exposure (Risks associated with specific job roles (e.g., healthcare, farming, agriculture etc))   - Exposure in homes and communities (Infection risks from living conditions, shared spaces, and hygiene practices, household works etc.) |
| Factors influencing Antimicrobial Use | - Education and Awareness (Knowledge about AMR and its sources, and attitude and practice) - Non-prescribed antibiotic use and sources - Access/ choice to healthcare and resources (availability and accessibility). - Distribution of labor, practices, and role (Household level) - Decision-making power and Autonomy - Prescription pattern. |
| Current relevant documents | - National and regional NAP, strategy, framework, act, guideline, policies addressing the AMR situation in Bangladesh and Sri Lanka and gaps related to gender issues. |
