# Supplementary material for: Gender and equity within antimicrobial resistance: a scoping review of evidence and policy in Bangladesh and Sri Lanka
Source: BMC Public Health. 2026 Jun 4;26:2313. doi: 10.1186/s12889-026-27842-y (PMC13455460; doi:10.1186/s12889-026-27842-y)
Supplement: Supplementary file 3 [file 12889_2026_27842_MOESM12_ESM.docx]

**Glossary Items:**

**Gender equity:** The process of treating men and women equally. To maintain equity, it is frequently necessary to implement policies that make up for the social and historical disadvantages that keep men and women from competing on an even playing field. Equity is average. The outcome is equality.

**Health Equity:** Equity is the absence of unfair, avoidable, or remediable differences among groups of people, whether those groups are defined socially, economically, demographically, or geographically or by other dimensions of inequality (e.g. sex, gender, ethnicity, disability, or sexual orientation).

**Intersectionality:** Intersectionality is an analytical perspective that examines how different social stratifies interact to create different experiences of privilege, vulnerability, and/or marginalization. An intersectional perspective recognizes that the combination of various factors experienced at individual and group levels is influenced by societal processes and structures of power.

**Gender unequal:** Level 1 of the WHO gender-responsive assessment scale: perpetuates gender inequality

by reinforcing unbalanced norms, roles and relations and often leads to a hierarchal

imbalance and inequalities in the enjoyment of rights or opportunities.

**Gender-blind:** Level 2 of the WHO gender-responsive assessment scale: ignores gender norms, roles and

relations and very often reinforces gender-based discrimination. By ignoring differences

in opportunities and resource allocation for women, men and gender-diverse people,

such policies are often assumed to be so-called fair as they claim to treat everyone the

same.

**Gender-responsive:** Health actions are gender-responsive when they consider gender norms, roles and

inequalities with measures to actively reduce their harmful effects. The World Health

Organization (WHO) defines five levels of action in its gender-responsive assessment

scale with increasing value as follows: gender-unequal, gender-blind, gender-sensitive,

gender-specific and gender-transformative, the last two levels indicating gender

responsiveness.

**Gender sensitive:** Level 3 of the WHO gender-responsive assessment scale: considers inequality generated

by unequal gender norms, roles and relations but takes no remedial action to address it.

Usually indicates gender awareness.

**Gender-specific:** Level 4 of the WHO gender-responsive assessment scale: considers the specific needs

of women, men and gender-diverse people and specifically targets a group to achieve

certain goals or meet needs. Even when inequality generated by unequal gender norms,

roles and relations are considered and remedial action to address their harmful effects is

taken, this may not change underlying causes of gender differences (5).

**Gender transformative:** Level 5 of the WHO gender-responsive assessment scale: addresses the causes of gender

based health inequities by transforming harmful gender norms, roles and relations

through the inclusion of strategies to foster progressive changes in power relationships

between women, men and gender-diverse people; for example, cisgender and

transgender men and women, other transgender people, non-binary people and other

people with different gender identities.

**Intersectional gender analysis:** The process of analysing how gender power relations intersect with other social stratifiers

(such as sex, gender, class, race, education, ethnicity, age, geographic location, religion,

migration status, ability, disability and sexuality) to affect people’s lives, how they create

differences in needs and experiences, and how policies, services and programmes either

exacerbate or mitigate these differences adapted from.

Source: World Health Organization, 2010

**Reference:**

Gender mainstreaming for health managers: a practical approach. Participant's notes. Geneva: World Health Organization; 2011. (<https://apps.who.int/iris/bitstream/handle/10665/44516/9789241501064_eng.pdf?sequence=2>, accessed 06 October 2025)
