# Supplementary material for: Gender and equity within antimicrobial resistance: a scoping review of evidence and policy in Bangladesh and Sri Lanka
Source: BMC Public Health. 2026 Jun 4;26:2313. doi: 10.1186/s12889-026-27842-y (PMC13455460; doi:10.1186/s12889-026-27842-y)
Supplement: Supplementary file 4 [file 12889_2026_27842_MOESM1_ESM.docx]

**Additional File 1: complete search strings for each database**

**Key search terms for PubMed**

| SL No | Key word | Remarks | Result |
| --- | --- | --- | --- |
| 1 | ((((((((((((Antimicrobial resistance*) OR (Antibiotic resistance*)) OR (Drug resistance*)) OR (Microbial resistance*)) OR (AMR)) OR (Drug Uptake*)) OR (Medicine uptake)) OR (purchasing medicine)) OR (Self-medication)) AND ((((((((((Gender*) OR (Equity*)) OR (Male)) OR (Female)) OR (Man)) OR (Women)) OR (sex)) OR (Gender disparity*)) OR (Social inequity*)) OR (Social disparity*))) AND (((((((((((((((((((((((((((((((Vulnerable groups) OR (Women)) OR (Adolescents)) OR (Children)) OR (Elderly)) OR (Youth)) OR (Poor people)) OR (Homeless people)) OR (Slum Dwellers)) OR (slum settlements)) OR (Informal settlements)) OR (Marginalized people)) OR (Minorities)) OR (Unemployed)) OR (Low-income people)) OR (Daily wage laborers)) OR (Female-headed households)) OR (widows)) OR (Displaced people)) OR (migrant people)) OR (climate-induced displacement)) OR (Climate affected migrants)) OR (Frontline health workers)) OR (sex worker)) OR (Community pharmacist)) OR (Farmer)) OR (Animal husbandry)) OR (Transgender)) OR (women lead household)) OR (pregnant women)) OR (Caregivers))) AND (((((((((((((((((((Study approach) OR (Methodology)) OR (Framework analysis)) OR (survey questionnaire)) OR (guidelines)) OR (Policies)) OR (SoPs)) OR (Operational plan)) OR (National action plan)) OR (intervention)) OR (implementation)) OR (health-seeking behavior)) OR (decision making)) OR (Access to health-care facilities)) OR (prescription pattern)) OR (Antimicrobial-use)) OR (messaging)) OR (policies)) OR (intervention) OR (health practices))) AND (Bangladesh) | Bangladesh | 309 |
| 2 | ((((((((((((Antimicrobial resistance*) OR (Antibiotic resistance*)) OR (Drug resistance*)) OR (Microbial resistance*)) OR (AMR)) OR (Drug Uptake*)) OR (Medicine uptake)) OR (purchasing medicine)) OR (Self-medication)) AND ((((((((((Gender*) OR (Equity*)) OR (Male)) OR (Female)) OR (Man)) OR (Women)) OR (sex)) OR (Gender disparity*)) OR (Social inequity*)) OR (Social disparity*))) AND (((((((((((((((((((((((((((((((Vulnerable groups) OR (Women)) OR (Adolescents)) OR (Children)) OR (Elderly)) OR (Youth)) OR (Poor people)) OR (Homeless people)) OR (Slum Dwellers)) OR (slum settlements)) OR (Informal settlements)) OR (Marginalized people)) OR (Minorities)) OR (Unemployed)) OR (Low-income people)) OR (Daily wage laborers)) OR (Female-headed households)) OR (widows)) OR (Displaced people)) OR (migrant people)) OR (climate-induced displacement)) OR (Climate affected migrants)) OR (Frontline health workers)) OR (sex worker)) OR (Community pharmacist)) OR (Farmer)) OR (Animal husbandry)) OR (Transgender)) OR (women lead household)) OR (pregnant women)) OR (Caregivers))) AND (((((((((((((((((((Study approach) OR (Methodology)) OR (Framework analysis)) OR (survey questionnaire)) OR (guidelines)) OR (Policies)) OR (SoPs)) OR (Operational plan)) OR (National action plan)) OR (intervention)) OR (implementation)) OR (health-seeking behavior)) OR (decision making)) OR (Access to health-care facilities)) OR (prescription pattern)) OR (Antimicrobial-use)) OR (messaging)) OR (policies)) OR (intervention) OR (health practices))) AND (Sri Lanka) | Sri Lanka | 80 |

**Key search terms for Google Scholar (Bangladesh):**

For each search conducted in Google Scholar, we screened and included studies from the first 10 pages of results.

| **SL no** | **Search terms** |
| --- | --- |
| **Bangladesh** | |
| GS-1 | ("Antimicrobial resistance" OR "AMR" OR "Antibiotic resistance") AND ("Gender Equity" OR Social inequity") AND ("Vulnerable groups") AND ("Bangladesh") |
| GS-2 | ("Antimicrobial resistance" OR "AMR" OR "Antibiotic resistance") AND ("Gender Equity" OR Social inequity") AND ("Women" OR "children" OR "Mothers" OR "Adolescent" OR "Children under-5" OR "Elderly") AND ("Bangladesh") |
| GS-3 | antimicrobial resistance Bangladesh Gender equity |
| Gs-4 | ("Antimicrobial resistance" OR "AMR" OR "Antibiotic resistance") AND ("Gender Equity" OR Social inequity") AND (" Poor OR homeless OR Slum dwellers OR Low income people") AND ("Bangladesh") |
| GS-5 | antimicrobial resistance Bangladesh |
| GS-6 | Health-seeking behavior & Access to health-care facilities AMR Bangladesh |
| GS-7 | Prescription pattern, antimicrobial- use and messaging in Bangladesh |
| GS-8 | Gender equity regarding prescription pattern, antimicrobial- use and messaging in Bangladesh |
| GS-9 | Antimicrobial resistance, gender equity Policies, National Action Plan, & intervention in Bangladesh |
| **Sri Lanka** | |
| GS-1 | ("Antimicrobial resistance" OR "AMR" OR "Antibiotic resistance") AND ("Gender Equity" OR Social inequity") AND ("Vulnerable groups") AND ("Sri Lanka") |
| GS-2 | ("Antimicrobial resistance" OR "AMR" OR "Antibiotic resistance") AND ("Gender Equity" OR Social inequity") AND ("Women" OR "children" OR "Mothers" OR "Adolescent" OR "Children under-5" OR "Elderly") AND ("Sri Lanka") |
| GS-3 | antimicrobial resistance Sri Lanka Gender equity |
| GS-4 | ("Antimicrobial resistance" OR "AMR" OR "Antibiotic resistance") AND ("Gender Equity" OR Social inequity") AND (" Poor OR homeless OR Slum dwellers OR Low income people") AND ("Sri Lanka") |
| GS-5 | antimicrobial resistance Sri Lanka |
| GS-6 | Health-seeking behavior & Access to health-care facilities AMR Sri Lanka |
| GS-7 | Prescription pattern, antimicrobial- use and messaging in Sri Lanka |
| GS-8 | Gender equity regarding prescription pattern, antimicrobial- use and messaging in Sri Lanka |
| GS-9 | Antimicrobial resistance, gender equity Policies, National Action Plan, & intervention in Sri Lanka |

**Key search terms for Scopus:**

| Sl. | Search terms | Remarks | Search results |
| --- | --- | --- | --- |
| 1 | TITLE-ABS-KEY(("Antimicrobial resistance*" OR "Antibiotic resistance*" OR "Drug resistance*" OR "Microbial resistance*" OR "AMR" OR "Drug Uptake*" OR "Medicine uptake" OR "purchasing medicine" OR "Self-medication") AND ("Gender*" OR "Equity*" OR "Male" OR "Female" OR "Man" OR "Women" OR "sex" OR "Gender disparity*" OR "Social inequity*" OR "Social disparity*") AND ("Vulnerable groups" OR "Women" OR "Adolescents" OR "Children" OR "Elderly" OR "Youth" OR "Poor people" OR "Homeless people" OR "Slum Dwellers" OR "slum settlements" OR "Informal settlements" OR "Marginalized people" OR "Minorities" OR "Unemployed" OR "Low-income people" OR "Daily wage laborers" OR "Female-headed households" OR "widows" OR "Displaced people" OR "migrant people" OR "climate-induced displacement" OR "Climate affected migrants" OR "Frontline health workers" OR "sex worker" OR "Community pharmacist" OR "Farmer" OR "Animal husbandry" OR "Transgender" OR "women lead household" OR "pregnant women" OR "Caregivers") AND ("Study approach" OR "Methodology" OR "Framework analysis" OR "survey questionnaire" OR "guidelines" OR "Policies" OR "SoPs" OR "Operational plan" OR "National action plan" OR "intervention" OR "implementation" OR "health-seeking behavior" OR "decision making" OR "Access to health-care facilities" OR "prescription pattern" OR "Antimicrobial-use" OR "messaging" OR "policies" OR "intervention" OR "health practices") AND ("Bangladesh")) | Bangladesh | 86 |
| 2 | TITLE-ABS-KEY(("Antimicrobial resistance*" OR "Antibiotic resistance*" OR "Drug resistance*" OR "Microbial resistance*" OR "AMR" OR "Drug Uptake*" OR "Medicine uptake" OR "purchasing medicine" OR "Self-medication") AND ("Gender*" OR "Equity*" OR "Male" OR "Female" OR "Man" OR "Women" OR "sex" OR "Gender disparity*" OR "Social inequity*" OR "Social disparity*") AND ("Vulnerable groups" OR "Women" OR "Adolescents" OR "Children" OR "Elderly" OR "Youth" OR "Poor people" OR "Homeless people" OR "Slum Dwellers" OR "slum settlements" OR "Informal settlements" OR "Marginalized people" OR "Minorities" OR "Unemployed" OR "Low-income people" OR "Daily wage laborers" OR "Female-headed households" OR "widows" OR "Displaced people" OR "migrant people" OR "climate-induced displacement" OR "Climate affected migrants" OR "Frontline health workers" OR "sex worker" OR "Community pharmacist" OR "Farmer" OR "Animal husbandry" OR "Transgender" OR "women lead household" OR "pregnant women" OR "Caregivers") AND ("Study approach" OR "Methodology" OR "Framework analysis" OR "survey questionnaire" OR "guidelines" OR "Policies" OR "SoPs" OR "Operational plan" OR "National action plan" OR "intervention" OR "implementation" OR "health-seeking behavior" OR "decision making" OR "Access to health-care facilities" OR "prescription pattern" OR "Antimicrobial-use" OR "messaging" OR "policies" OR "intervention" OR "health practices") AND ("Sri Lanka")) | Sri Lanka | 10 |

**Key Search Term for IMSEAR:**

| **Key Search Term** | **Result** |
| --- | --- |
| Bangladesh | |
| (Antimicrobial resistance) AND (Bangladesh) | 35 |
| (Antimicrobial resistance) OR (AMR) OR (Antibiotic resistance) AND (Bangladesh) | 53 |
| (Antimicrobial use) OR (Antibiotic use) AND (Bangladesh) | 24 |
| (Prescription Pattern) AND (Antibiotic) AND (Bangladesh) | 5 |
| (Health-seeking behavior) OR (Access to health-care facilities) OR (Antimicrobial resistance) AND (Bangladesh) | 28 |
| (Antibiotic Use) OR (Health-seeking behavior) OR (Healthcare access) AND (Bangladesh) | 17 |
| Sri Lanka | |
| (Antimicrobial resistance) AND (Sri Lanka) | 9 |
| (Antimicrobial resistance) OR (AMR) OR (Antibiotic resistance) AND (Sri Lanka) | 10 |
| (Antimicrobial use) OR (Antibiotic use) AND (Sri Lanka) | 10 |
| (Prescription Pattern) AND (Antibiotic) AND (Sri Lanka) | 0 |
| (Health-seeking behavior) OR (Access to health-care facilities) OR (Antimicrobial resistance) AND (Sri Lanka) | 10 |
| (Antibiotic Use) OR (Health-seeking behavior) OR (Healthcare access) AND (Sri Lanka) | 6 |
