# Supplementary material for: Gender and equity within antimicrobial resistance: a scoping review of evidence and policy in Bangladesh and Sri Lanka
Source: BMC Public Health. 2026 Jun 4;26:2313. doi: 10.1186/s12889-026-27842-y (PMC13455460; doi:10.1186/s12889-026-27842-y)
Supplement: Supplementary file 5 [file 12889_2026_27842_MOESM2_ESM.docx]

**Summary statistics:**

**Total studies:** 38

**Study types:**

- Quantitative: 31

- Qualitative: 5

- Mixed methods: 2

**Settings:**

- Urban: 23

- Rural: 10

- Both: 5

**Themes covered (with count):**

1. Gender-based knowledge, attitude, and practice regarding AMR: 16
2. Gender disparities in non-recommended antibiotic use and sources: 13
3. Influence of gender roles and norms in AMR: 9
4. Gender-based vulnerability to infection and exposure to AMR: 7
5. Role of Healthcare Providers and Prescription Patterns: 5

**Additional File 2: Summary of findings for included papers from Bangladesh**

| **SL no** | **Citations** | **Study type** | **study settings** | **Theme/sub-theme covered** | **Key findings** |
| --- | --- | --- | --- | --- | --- |
| 1 | (Alam, M. J., et al, 2019) | Retrospective | Urban  Laboratory data (3765 urine samples) was collected from the Microbiology Department of a diagnostic center, Dhaka, Bangladesh, from 1 July 2017 to 30 June 2018. | Vulnerability to infection | 1. Escherichia coli was the most common pathogen (83.8%) in urinary tract infections (UTIs), which were more common in males aged ≤10 and >60 years and more common in females aged 10 to 60 years.  2. In contrast to other Uro pathogens, which showed varying sensitivity patterns across genders, E. coli isolates that were 50.7% male and 41.6% female exhibited resistance to cefotaxime. |
| 2 | (Alam, M. U., et al, 2019) | Mixed method (In-depth interviews and structured observation) | Rural & Urban.  Rural: 8 rural households from 8 villages in Mirzapur sub-district, 15 commercial poultry farms from 10 villages in Mirzapur  Urban: 5 live bird markets from Dhaka   Data was collected from poultry farmers in rural villages for traditional animal husbandry, commercial poultry farms in peri-urban sites around Dhaka, live poultry markets in high-density populated areas of urban Dhaka. | Gender roles and norms (Occupational exposure) | 1.Poor hygiene, a lack of safety equipment, and inappropriate waste management of poultry, particularly by rural women and male farm and market workers, increase the risk of AMR in Bangladesh. |
| 3 | (Bepari et al, 2023) | Cross-sectional | Rural (Sylhet and Jashore)  Data was collected from all male participants (396 pharmacy shopkeepers and unqualified village medical practitioners) aged ≥ 18 years and living in Sylhet and Jashore districts in Bangladesh between June–December 2021. | Knowledge, attitude, practice regarding Antibiotic use | 1.Unqualified male healthcare professionals in rural Bangladesh (62%) had moderate to poor KAP on antibiotics, which exacerbated the risks of AMR because of illogical prescribing and a lack of proper infrastructure. |
| 4 | (M. Biswas et al, 2014) | Cross-sectional prospective study | Rural (Jessore Sadar, Manirampur, and Keshabpur upazilas in Jessore district, Khulna)  A total of 900 prescriptions were analyzed during March 2013 to June, 2013 from the patient’s prescription and by directly interviewing the patients who were prescribed at least one antibiotic in three adjacent cities of Jessore district under Khulna Division Bangladesh. | Role of healthcare providers and Prescription pattern | males were prescribed 22% more antibiotics than females, |
| 5 | (M. Biswas et al, 2014) | Cross-sectional | Urban, Rajshahi city  Self-medication of antibiotic data (1300) was collected from different locations of Rajshahi city between March to April, 2014. | Self-medication and source | Males (83.57%) tended to use more self-medicated antibiotics than females.  Females are more cautious and sensitive to their lives rather than males and also for self-medication of antibiotics. |
| 6 | (Chapot et al, 2021) | Cross-sectional | Urban  To investigate KAP regarding antibiotic use and AMR, data was collected from a total of 3002 veterinary and non-veterinary students from 12 public universities in Bangladesh between November 2019 to March 2020. | Knowledge, attitude, practice regarding Antibiotic use | Gender influence knowledge regarding antibiotic use and resistance related among veterinary students and non-medical students and females are more knowledgeable than males. |
| 7 | (Chowdhury et al, 2019) | Qualitative study | Rural, Matlab  The study was conducted in a rural community, Matlab, Chandpur District of Bangladesh between 10 March and 6 April 2017 through 6 focus group discussions and 16 in-depth interviews. | Knowledge, attitude, practice regarding Antibiotic use  self-medication and source  Gender-based roles and norms (decision-making and access to healthcare  Role of healthcare providers and Prescription pattern | 1.Females are more dependent on their husband’s decision for health care access.  2. They prefer to go to nearby pharmacies for better accessibility and social network.  3. Prefer to take an extra dose of antibiotic for quick recovery. |
| 8 | (Darj et al, 2019) | Qualitative (In depth interview) | Urban, Dhaka  A total of 24 male retail Pharmacist’s knowledge and perception regarding self-medication, antibiotic use was examined through In-depth interviews in Dhaka city. all of the participants were male in 2018. | Knowledge, attitude, practice regarding Antibiotic use  Self-medication, access to healthcare,  Role of healthcare providers and Prescription pattern | 1.People of rural areas are more AMR susceptible because of the poor quality of medicine provided in the pharmacy compared to urban areas.  2. Self-medication and incomplete doses of antibiotics is more common when symptoms improve, especially in less critical conditions, due to cost and time. |
| 9 | (Do et al, 2021) | Mixed method (quantitative and qualitative) | Rural & Urban both (Bangladesh: Rural)  Antibiotic access and use in six LMICs across Africa (Mozambique, Ghana, and South Africa) and Asia (Bangladesh, Vietnam, and Thailand) over a 2·5-year study period (July 1, 2016–Dec 31, 2018). | Knowledge, attitude, practice regarding antibiotic  Gender roles and norms (decision-making and access to healthcare) | 1.Female are less likely to have knowledge regarding AMR than male. 2. Male respondents share medicine and also advice to take antibiotics to their near and dear one. |
| 10 | (Garbern et al, 2021) | Quantitative (Secondary data analysis) | Urban  MDR pathogens test among the 1198 children aged> 5 years, adults, and elderly patients with acute diarrhea at a hospital in Dhaka between March 2019 and March 2020. | Vulnerability to infection | Females are more susceptible to multi drug resistance compared to male. |
| 11 | (Ghosh et al, 2023) | Cross-sectional | Urban and rural  Data from a nationwide multiple indicators cluster survey (MICS), 2019 was used to find the factors related to infectious disease and antibiotic use among 23,099 children. | Knowledge, attitude, practice regarding Antibiotic use | 1. Caregivers (mother) of the children had a significant impact on the misuse of antibiotics for their children.  2. Mother’s education and household wealth were significantly associated with the reported excessive antibiotic usage. |
| 12 | (Hassan et al, 2021) | Cross-sectional | Urban  KAP regarding AMR of 420 male poultry farmers (broiler and layer farmers) was investigated from the seven districts in Bangladesh using a tested and paper-based questionnaire. | Knowledge, attitude regarding Antibiotic use | 1.Among the poultry farmers, knowledge is very low regarding using antibiotics.  2.One-third of the total farmers depend on themselves or others for the usage of antimicrobials rather than any registered vets. |
| 13 | (Hossain et al, 2023) | Cross sectional | A web-based survey was conducted to find out the irrational use of antibiotics among the university teachers and students (n=783) during January 1 to February 28, 2022. | Self-medication and source | 1.Females were more (66% higher) at risk of developing antibiotic resistance (ABR) than males.  2. The primary reason for self-medication is previous experience. |
| 14 | (Islam et al, 2022) | Cross-sectional | Urine samples were prospectively collected from 4,500 patients at a diagnostic clinic in Dhaka, Bangladesh during 2016-2018 to estimate the prevalence of community-acquired UTI caused by antibiotic resistant organisms. | Vulnerability to infection | 1. Women were more likely to have UTI compared to males. Females patients had 1.48 times higher prevalence of UTIs than males, while the male patients (73%), especially the children were more infected with multidrug-resistant (MDR) organisms.  2. A significantly higher proportion of male patients (73%) tested positive for MDR  organisms compared to females (63%).  3. The prevalence of MDR-UTIs did not differ significantly among the patients' age groups.  4. Among the children, the male children had 72% higher odds of being infected with MDR organisms than females. |
| 15 | (Islam, M. R. et al, 2021) | Quantitative | A total of 5279 infants aged 2–6 months in a hospital in Dhaka, Bangladesh to investigate severe acute malnutrition and history of antibiotic use, between September 2018 and June 2019. | Prescription pattern | 1.The prevalence of prescribing antibiotics is higher among the male (52.4%) compared to females (51.4%). |
| 16 | (Islam, M. W. et al, 2024) | cross-sectional study | Contributing factors to antibiotic misuse among 704 parents of school going children for assessing their knowledge, attitude, and practice (KAP) in Dhaka South City between August 20 to August 30, 2022. | Knowledge, attitude, practice regarding Antibiotic use | In terms of positive attitudes towards antibiotic resistance, male have 140% higher odds than their counterparts. |
| 17 | (H. Kabir et al, 2022) | Cross-sectional | Antibiotic self-administration among 295 adults who used antibiotics two months preceding the survey in Bangladesh. | Self-medication and source | Self-medication was higher among females (21.43%) than males (14.18%). |
| 18 | (Kalam et al, 2021) | Cross-sectional | KAP and prescription of antibiotic use among the vets by the veterans working in different veterinary hospitals in different Upazilas in Bangladesh between April 2021 to May 2021. | Knowledge, attitude regarding Antibiotic use | 1.Among the veterinary practitioners, 83.3% (n = 363) were male and 16.7% (n = 73) were female.  2.Gender is not associated with the knowledge and attitude of AMR.  3. Males are more likely to practice AMR compared to female (OR: 0.59). |
| 19 | (Kalam et al, 2022) | Qualitative (semi-structured telephone interviews) | Antibiotic-seeking behaviors among the 40 COVID 19 patients who took care at home in Dhaka and Chattogram, Bangladesh between May and June 2021. | Self-medication and source | 1.During COVID-19, females were more likely to seek antibiotics right away after testing positive, while the males tended to rely more on previous personal experiences or prescriptions.  2.Self-management practices were same for both gender, women prioritize their treatment, whereas men frequently put off treatment until symptoms worsened or treatments didn't work. |
| 20 | (Khanam R. et al, 2017) | Cross sectional | Data was collected from 371 streets and 329 residence female sex workers (FSWs) receiving HIV prevention services at 24 drop-in centers in Dhaka between August and October 2014. | Gender roles and norms (Occupational exposure) | 1.Among the female sex workers, 11.1% had a STI, with chlamydia being the most common (6.3%).  2.In N. gonorrhoeae, antibiotic resistance was reported for azithromycin (21.4%) and cefixime (7.1%). |
| 21 | (Khanam U. S. et al, 2018) | cross-sectional observational study | Prescription pattern of 242 patients from Debidwar Upazila of Comilla District, Bangladesh were analyzed. | Role of healthcare providers and Prescription pattern | 1.Ciprofloxacin was prescribed to 30.17% patients (Male: 21.90%, Female: 8.26%) for common cold.  2. Only 23.55% patients were recommended by the doctors for diagnosis tests before prescribing any anti-bacterial agents.  3. For 81.81% cases, physicians prescribed antibiotics by his/her own assumption. |
| 22 | (Lee A. C. et al, 2019) | Population-based cluster randomized controlled trial (cRCT) | Urine specimens were collected at the household level in 4242 pregnant women (< 20 weeks gestation) for culture and antibiotic susceptibility testing in Sylhet, Bangladesh between January 2, 2012 and July 28, 2015. | Vulnerability to infection | 1.About 8.9% of pregnant women had UTIs in Sylhet, Bangladesh.  2. E. coli was the most prevalent pathogen (38%).  3. Only 67% of E. coli were sensitive to third-generation cephalosporins, indicating substantial antibiotic resistance. |
| 23 | (Mannan et al, 2024) | Cross-sectional | Self-medication with antibiotics (SMA) among different indigenous group populations aged 18 years or more orders residing in the three districts of CHT (Rangamati, Bandarban, and Khagrachari) between January to early July 2021. | Self-medication and source | 1.Men had 1.6 times higher odds of self-medication of antibiotic than women.  2Self-medication for antibiotic usage was significantly different by education level, occupation, and family income along with gender. |
| 24 | (Marzan et al, 2021) | Cross-sectional | KAP concerning antibiotic use and resistance among students at Jahangirnagar University (JU), Bangladesh between January to April 2020. | Knowledge, attitude, practice regarding Antibiotic use  self-medication and source | 1.Among the university Students, knowledge about antibiotics resistance and it's uses is found 69% higher among the male compared to female. |
| 25 | (Nizame et al, 2021) | Qualitative study (co-design workshops, IDI, KII,  stakeholder meeting) | Policy awareness related data was collected from 4 co-design workshops and 24 IDI with medicine shop operators and customers, 12 KII with key personnel involved in aspects of the antibiotic supply chain and a group discussion with stakeholders representing key actors in informal market systems namely: representatives from the government, private sector and other organizations and selected area was one (Kaliganj sub-district of Gazipur District) and one urban area (Rupganj sub-district of Narayanganj District) between June 2019 to August 2020. | Knowledge, attitude, and practice regarding Antibiotic use | In Bangladesh context, most of the drug seller are male, their knowledge related to AMR and its national policy is very low. |
| 26 | (Roess et al, 2015) | Cross-sectional | Human and agricultural antibiotic use and resistance in households in rural Sylhet district, Bangladesh between July 2002 and December 2004. | Gender based roles and norms (occupational exposure) | 1.For household cheers and care giving positions for both poultry and child, women were most vulnerable for AMR.  2. It was also common practice to keep animals in cramped place and to fertilize with animal manure and use shared water sources towards AMR. |
| 27 | (Rousham et al, 2023) | Observational study | Customer's gender, antibiotic purchases, and prescription use were observed in 20 drug shops in one rural area of Mirzapur  sub-district (Upazila) in Tangail district and the urban town center in Tongi sub-district, Gazipur district between October-November 2017 | Role of healthcare providers and Prescription pattern  Gender roles and norms (decision-making and access to healthcare) | 1.Compared to rural places, women customers in medicine shops were more common in urban places (47.1% in urban and 17.2% in rural).  2.For pursing the antibiotic using antibiotic was more likely among women than men (OR: 4.04) and more likely among urban compared to rural customers (OR: 4.31).  3.For animal medicine, the rate of women customer was relatively low (10.9%). |
| 28 | (Saha et al, 2022) | Cross-sectional | The practice of self-medication among the indigenous population living in Chittagong Hill Tract (Rangamati, Bandarban, and Khagrachari) were observed between October to early December 2020. | Self-medication and source  Gender roles and norms (access to healthcare, occupational exposure) | 1.The practice of self-medication was high among the male (57.6%) than female (41.9%).  2. About 80.9% of the total respondent took self-medicated antibiotic.  3. Female participants were 46% less likely to take medications without prescriptions than their male counterparts.  4.People involved with agricultural work used more antibiotics than service.  5.The primary source self-medicated antibiotic was the local pharmacy. |
| 29 | (Saha et al, 2023) | Cross-sectional | Self-medication on antibiotic use data was collected from two cities Thimphu, Bhutan (275) and Chattogram, Bangladesh (723). between May and early December 2019. | Self-medication and source | 1.The rate of self-medication is high among the female in Chattogram (55.7%) compared to male (44.3%). Among them, about 60% people consume antibiotic as a self-medication. |
| 30 | (Sarker et al, 2024) | Cross-sectional | KAP of antibiotic use and resistance data collection was conducted among the registered veterinary practitioners (208) of Bangladesh listed by the Bangladesh Veterinary Council (BVC) between August to September 2020. | Knowledge, attitude, practice regarding Antibiotic use  Gender roles and norms (occupational exposure) | 1.Male veterinarians had a significantly 2.37 times higher knowledge score compared to female veterinarians.  2.Gender distribution indicated that a lower number of female veterinarians engaged in clinical practice than male. |
| 31 | (Seam et al, 2018) | Cross-sectional | Practice of self-medication of antibiotic among undergraduate pharmacy students (250) in five repuFile public universities of Bangladesh between January to June 2016. | self-medication and source | 1.Self-medication was nearly same among male and female students (45.20% and 42.80%, respectively), with no significant difference.  2.About 15.60% (39) of the total students self-medicated with antibiotics; the percentage is higher among males (10.80%) than females (4.80%), but it is not statistically significant. |
| 32 | (Tanni et al, 2021) | Cross-sectional | Drug susceptibility test was done using 1000 clinical isolates collected from two different hospitals of Chattogram to detect KPN’s response to 16 types of antibiotics between August 2018 to November 2019. | Vulnerability to infection | 1.In Klebsiella pneumoniae isolates, high levels of multidrug resistance were seen among female than male.  2.Females were more likely to carry the blaNDM-1 gene (52.3%), but males were more likely to carry the uge and blaSHV-11 genes (51.17% and 55.3%, respectively). |
| 33 | (Tasmim et al, 2023) | Qualitative survey | Rational use of antibiotic among the 140 investigated farmers, 50 were from Gazipur district, 30 were from Kishoreganj district and 60 were from Mymensingh district in Bangladesh investigated between March to May 2019. | self-medication and source  Knowledge, attitude regarding Antibiotic use | 1.Compared to male farmers, female were 4.5 times more likely to misuse antibiotics in poultry as a result of having less access to education and awareness campaigns.  2. Male farmers showed more knowledge and practices because they were more exposed to information on antibiotic use and resistance. |
| 34 | (Yasmin et al, 2018) | Cross-sectional observational study | KAP regarding antibiotic among the patients (562) attending the OPD of a Hospital, in Dhaka city, Bangladesh. in February, 2017 were observed. | Knowledge, attitude, practice regarding Antibiotic use | 1.Female respondents had better knowledge regarding AMR compared to male, while there was no significant gender difference for practicing it. |
| 35 | (Hossain, A. et al, 2020) | cross sectional | The sensitivity to amikacin, cefixime, ciprofloxacin, and other antibiotic were determined for 1663 E. coli isolates obtained from various outlets of a medical service in Bangladesh between November 2017 until August 2018. | Vulnerability to infection | 1.The male isolates are 2.27 times more resistant to amikacin than females.  2.Resistant to colistin & nitrofurantoin among the male isolates were 2.09 times and 1.45 time more resistant than females.  3.For both genders, meropenem and imipenem were found similarly effective. |
| 36 | (Nuzhat, S. et al, 2022) | Survellance study | Stool specimen was collected from all enrolled patients and tested clinical microbiology laboratory in Dhaka/ Matlab for the diagnosis of antimicrobial resistance in shigellosis between January 2001 and December 2020. | Vulnerability to infection | Gander had no significant impact on antibiotic resistance in shigellosis among under-5 children in both urban and rural sites with the changes of time. |
| 37 | (Wahab, A. et al, 2023) | Cross-sectional | KAP regarding antibiotic use and resistance among the three university students (1000) in January, 2022 were investigated. | Knowledge, attitude, practice regarding Antibiotic use  self-medication and source | 1.Gender is significantly associated with self-medication with antibiotic, female students (77.2%) and male (50%). 2. Women had a greater percentage of inadequate knowledge about AMR (59.9%) than men (50.9%) and the practice (45.3%) than men (30.7%). |
| 38 | (Hicks, J.P. et al, 2021) | Cross-sectional survey | KAP and practice regarding antibiotic practices among household members and their domestic animals within rural communities in Cumilla district in Bangladesh (682 female and 620 male household heads) between May and June 2018. | Knowledge, attitude, practice regarding Antibiotic use  self-medication and source | 1.Men and women were aware of antibiotic use (70%) but both genders were involved in the misuse of antibiotics, such as using antibiotics for colds or coughs (52%).  2.The rate of prescription uses and full dose completion was higher among the male their counterpart.  3.Compared to men, women were more prone to abuse antibiotics in animals for prophylactic or growth goals. |
