# Supplementary material for: Gender and equity within antimicrobial resistance: a scoping review of evidence and policy in Bangladesh and Sri Lanka
Source: BMC Public Health. 2026 Jun 4;26:2313. doi: 10.1186/s12889-026-27842-y (PMC13455460; doi:10.1186/s12889-026-27842-y)
Supplement: Supplementary file 6 [file 12889_2026_27842_MOESM3_ESM.docx]

**Summary statistics:**

**Total studies:** 22

**Study types:**

- Quantitative: 22

- Qualitative: 0

- Mixed methods: 0

**Settings:**

- Urban: 13

- Rural: 2

- Both: 3

- Unspecified: 4

**Themes covered (with count):**

1. Gender-based knowledge, attitude, and practice regarding AMR: 7
2. Gender disparities in non-recommended antibiotic use and sources: 4
3. Influence of gender roles and norms in AMR: 2
4. Gender-based vulnerability to infection and exposure to AMR: 8
5. Role of Healthcare Providers and Prescription Patterns: 1

**Additional File 3: Summary of findings for included papers from Sri Lanka**

| **SL no** | **Author name, [Ref]** | **Study Type** | **Study settings** | **Theme/Sub-theme** | **Key findings** |
| --- | --- | --- | --- | --- | --- |
| 1 | (Abhayasinghe et al, 2021) | Cross -sectional | A self-administrated web-based questionnaire was responded by the 204 internet users (females 120, males 84) about their knowledge, attitude and practice of antibiotic use from November 2019. | Knowledge, attitude, practice regarding antibiotic use | The mean score of knowledge about AMR is high among the female (5.1) participants compared to male (4.6). |
| 2 | (Amarasinghe et al, 2021) | Cross-sectional | The knowledge about consequences of inappropriate antibiotic use, ineffective treatment data were collected using a self-administered questionnaire by approaching all the eligible pharmacists (N=100) in community and hospital pharmacists in Galle District, Sri Lanka. | Knowledge, attitude regarding antibiotic use | 1.The socio-demographic factors did not have any impact on the regarding knowledge, except gender.  2. Males were 0.36 times less likely to have high level of knowledge related to AR than females. |
| 3 | (Dilrukshi et al, 2021) | Cross-sectional | Two low vaginal and rectal swabs were collected from 100 pregnant women from four teaching hospitals in Western Province, Sri Lanka to test several antibiotic sensitivities from January to April 2019. | Vulnerability to infection | In Western Province hospitals, Sri Lanka, GBS colonization was found among 18% and 49% of pregnant women >35 weeks of gestation using culture and RT-PCR, respectively.  GBS isolates were sensitive to most antibiotics but showed resistance to erythromycin (5/18) and clindamycin (4/18). |
| 4 | (Dilrukshi et al, 2023) | Cross-sectional | To test antibiotic susceptibility and characterized antibiotic resistance genes of group B Streptococcus (GBS) isolates, data was collected from 175 pregnant women of 35 weeks of gestation attending antenatal clinics in four teaching hospitals in Srilanka from January 2019 to July 2019. | Vulnerability to infection | All isolates were sensitive to penicillin.  For group B Streptococcus, resistance to erythromycin and clindamycin had increasing rates. |
| 5 | (Elwitigala et al, 2023) | Cross-sectional | Blood sample was collected from 194 people living with HIV newly initiating ART in each of the 18 selected ART facilities in Srilanka from January 2021 to July 2021. | Vulnerability to infection | The prevalence of pretreatment drug resistance (PDR) to efavirenz/nevirapine was higher among women (14.1%) than men (7.0%), though the difference was not statistically significant. |
| 6 | (Gunawardhana et al, 2015) | Cross-sectional | The practice of self-medication of antibiotic among the university students whose consumption of antibiotics with their experience in the past six months before the survey were examined. | Knowledge, attitude, practice regarding antibiotic use  self-medication and source | 1.The most frequently used antibiotic was amoxicillin (95.4%).  2. There was a significant difference in knowledge on antibiotics between health science and non-health science students.  3. There is no significant difference between the knowledge of male and female student. |
| 7 | (Jayawardhana et al, 2023) | Cross-sectional | The knowledge and practice of antibiotic use among the 347 medical students in Sri Lanka was observed during a one-month period from 24/05/2021 onwards. | Knowledge, attitude regarding antibiotic use  self-medication and source | 1.The practice and knowledge of antibiotic use and resistance did not differ significantly with gender.  2. Antibiotic misuse patterns were associated with gender, year of study, having a health care worker at home and knowledge score. |
| 8 | (Jayaweera & Kumbukgolla, 2017) | Cross- sectional | Nasal and axillary swabs were collected from livestock farmers (livestock, n=188 and related farmers n=94) community in Anuradhapura District of Sri Lanka in January-March 2017 to evaluate methicillin-susceptible Staphylococcus aureus (MRSA) colonization and its antimicrobial resistance pattern. | Gender roles and norms (Occupational exposure) | 1.Males were significantly more involved in livestock farming than females (p=0.021).  2.Pig farming showed the highest MRSA prevalence (26.6%) and a 2.4 times higher risk of transmission between animals and humans compared to cattle and poultry farming. |
| 9 | (Kumarasinghe & Weerasinghe, 2024) | Cross- sectional | Practice of oral pediatric antibiotic suspensions (OPAS) among 835 primary caregivers of 3–5-year-old children from Child welfare clinics in Ratnapura District of Sri Lanka. | Gender roles and norms (Occupational exposure) | 1.Parent's gender were significantly associated with the performance in reconstituting the oral pediatric antibiotic suspension of their children along with level of education, and geographical region.  2. Male caregivers showed lower capability compared to females, influenced by factors like education and the presence of preschool-aged children or older siblings (p = 0.002). |
| 10 | (Meredith et al, 2021) | Cross- sectional | Rectal swabs were collected from women and infants at enrollment and 4–6 weeks later from a obstetric/gynecologic wards of a hospital in Southern Province, Sri Lanka, from August 2016 to January 2017 for intestinal colonization with multidrug-resistant Enterobacteriaceae (MDRE) | Vulnerability to infection | 1.Compared to newborns (1.5%), mothers (12–17.6%) had a greater prevalence of MDRE colonization.  2. Infant's colonization had increased to 15.9% by six weeks of their birth.  3. There was no other significant risk factors found, however mothers who had more children at home had a higher chance of developing MDRE colonization (p = 0.001). |
| 11 | (Munasinghe et al, 2021) | Cross- sectional | A self - collected nasal swab and a peri-rectal swab was collected from 322 undergraduate students between 18 to 28 years of age to identify the rates of asymptomatic colonization with selected antibiotic-resistant organisms between December, 2017 to October 2018. | Vulnerability to infection | MRSA colonization was substantially greater in males (5.8%) compared to females (3.3%), While there is no significant difference between the ESBL colonization rates across males (15.4%) and females (12.7%). |
| 12 | (Nanayakkara et al, 2018) | Prospective observational study | A low vaginal swab was collected from (250) mothers and a peri-rectal swab was collected from the (159) neonates by a Professorial Obstetrics Unit of a hospital in Sri Lanka to identify the colonization rates for Escherichia coli, Klebsiella spp. and Streptococcus agalactiae between August, 2015 to January 2016. | Vulnerability to infection | Among 250 pregnant women, pre-delivery colonization rates for MDR Klebsiella spp. or E. coli were 0.4%, while there were no significant differences with post-delivery rates. |
| 13 | (Rathish & Wickramasinghe, 2020) | Cross- sectional | A community-based data based on the antibiotic self-medication during the last 3 months of the study were collected among dwellers of Anuradhapura, Sri Lanka December 2019. | Self-medication and source | The prevalence of antibiotic self-medication was 2.6%, 2.5% (5/203) in women and 2.8% (5/181) in men and there was no statistically significant association between genders and self-medication. |
| 14 | (Samaranayake et al, 2019) | Cross-sectional | Data was collected from 100 non-repetitive MRSA isolates from the microbiology laboratory, National Hospital of Sri Lanka (NHSL) from November 2013 to March 2014 to test the antimicrobial susceptibility of CA and HA MRSA strains. | Vulnerability to infection | 1.In terms of patient’s genders, there was no significant difference in acquiring CA MRSA and HA MRSA, along with age and ethnicity.  2. CA-MRSA isolates were significantly more sensitive to ciprofloxacin, fusidic acid, tetracycline, cotrimoxazole, and gentamicin. |
| 15 | (Sandaruwan & Dissanayake, 2022) | Cross-sectional | Knowledge and awareness related to antibiotic resistance and prescription behavior of companion animal among the 102 veterinary practitioners in Sri Lanka were collected using a web-based questionnaire. | Prescription pattern | 1.Among the veterinarian practitioners, there was no significant difference in the prescription behavior among males and females.  2. Also, there was no significant association of awareness with gender. |
| 16 | (Jayawardena et al, 2022) | Cross-sectional | Knowledge on antibiotic use and practices related to antibiotic prescribing among 262 participants from various fields of Medicine working in Sri Lanka were collected. | Knowledge and attitude regarding antibiotic | 1.There was no relation between knowledge, practice score and gender.  2. A significant association was observed among knowledge score and year of practicing. |
| 17 | (Subashini &. Udayanga, 2020) | Cross-sectional | Self-medication practice of antibiotic among 700 randomly selected undergraduates of three state universities in Sri Lanka were enrolled from February to April 2019. | Self-medication and source | Males had 1.65 times higher tendency for SM in comparison to female undergraduates, although the effect of gender on SM behavior was found to be insignificant |
| 18 | (Wijekoon et al, 2014) | Descriptive observational study | A total of 745 adult in-patients in a tertiary care hospital in Sri Lanka with positive urine culture and clinical features compatible  with UTI were enrolled for antibiotic sensitivity testing. | Vulnerability to infection | 1.Males and people over 65 had lower rates of antimicrobial susceptibility, and nitrofurantoin had the highest oral susceptibility despite its decreased effectiveness in these populations.  2. Antibiotics such as cefuroxime and ciprofloxacin were especially less likely to affect males (p<0.01). |
| 19 | (Zawahir et al, 2021) | Cross-sectional | A national level study was conducted to investigate the knowledge and attitudes toward antibiotic use and self-medication in 1100 randomly selected household in Sri Lanka. | Knowledge and attitude, practice regarding antibiotic | Female had 43% less tendency to take self-medication than male, while it was not significant |
| 20 | (Zhang et al, 2021) | Cross-sectional | Inappropriate antibiotic use among the 409 patients from outpatient Department of a public tertiary medical center in Southern Province, Sri Lanka from February to April 2019 were investigated. | Role of healthcare providers and Prescription pattern | 1.Amoxicillin was the most frequently prescribed agents and first-generation cephalosporins (38, 26.0%).  2. Respiratory illness was the most common reason for antibiotic use, following 66.1% of patients presenting with cough and 78.8% of those presenting with rhinorrhea or nasal congestion.  3. There is no association between gender and prescription pattern of antibiotic. |
| 21 | (Abhayasinghe et al, 2021) | Cross-sectional | Awareness and knowledge regarding antibiotics and antibiotic resistance among 450 patients who attended the  Outpatient Department of Teaching Hospital, Peradeniya, Sri Lanka were investigated. | Knowledge and attitude regarding antibiotic | The proportion of male patients giving the correct answer (n=36, 19.9%) did not  differ significantly from that of females (n=53, 19.7%). |
| 22 | (Gunasekera et al, 2022 | Cross-sectional | Knowledge, perception regarding antibiotics usage among the general public in two diverse Sri Lankan communities: ordinary urban (182) and indigenous rural (147) were investigated. | Knowledge and attitude, practice regarding antibiotic | Gender is not associated with the knowledge regarding antibiotic and also the ability of selecting it. |
