# Supplementary material for: Gender and equity within antimicrobial resistance: a scoping review of evidence and policy in Bangladesh and Sri Lanka
Source: BMC Public Health. 2026 Jun 4;26:2313. doi: 10.1186/s12889-026-27842-y (PMC13455460; doi:10.1186/s12889-026-27842-y)
Supplement: Supplementary file 7 [file 12889_2026_27842_MOESM5_ESM.docx]

**Narrative summary:**

A total of 10 grey documents were reviewed that addressed AMR. The key pattern of all documents is the same; none of them acknowledge the gender dimension, and all of the documents are Gender-blind because of common gaps across the documents, which are mentioned below:

1. Ignores gender norms, roles, and relations, with no requirement for gender-disaggregated AMR surveillance data
2. Very often reinforces gender-based discrimination, no attention to gender norms that influence antibiotic-related health-seeking behaviour, and vulnerability to infection.
3. Ignores differences in opportunities and resource allocation for women and men, especially, no discussion on gender based occupational exposure.
4. All constructed based on the principle of being “fair” by treating everyone the same, no gender-based communication or behaviour change strategies.

**Additional File 5: Policy documents and grey articles in Sri Lanka that addressed AMR**

| **Policy/ Guideline/ Framework** | **Year** | **AMR status** | **Gender Responsive Assessment Scale** |
| --- | --- | --- | --- |
| 1. National Strategic Plan for Combating Antimicrobial Resistance in Sri Lanka 2017 – 2022 ( https://www.who.int/publications/m/item/sri-lanka-national-strategic-plan-for-combating-antimicrobial-resistance-in-sri-lanka-2017-2022 ) | 2017 | Addressed | Gender Blind |
| 2. National Strategic Plan for combating AMR 2023-2028 ( <https://cdn.who.int/media/docs/default-source/antimicrobial-resistance/amr-spc-npm/nap-library/sri-lanka-national-strategic-plan-2023-2028.pdf?sfvrsn=d54326e0_3&download=true> ) | 2023 | Addressed | Gender Blind |
| 3. National Action Plan for Health Security of Sri Lanka 2019-2023 ( <https://extranet.who.int/sph/sites/default/files/document-library/document/NAPHS%20SRI%20LANKA%20-printed%20version.pdf> ) | 2019 | Addressed | Gender Blind |
| 4. Empirical and prophylactic use of antimicrobials- National guideline 2016 ( <https://slmicrobiology.lk/antibiotic-guidelines-2016/> ) | 2016 | Addressed | Gender Blind |
| 5. SLMA Antimicrobial Guideline 2024 ( <https://slma.lk/wp-content/uploads/2014/12/Guidelines-on-the-use-of-antimicrobial-agents.pdf> ) | 2024 | Addressed | Gender Blind |
| 6. Annual Health Bulletin 2021 ( <https://www.health.gov.lk/wp-content/uploads/2024/04/Annual-Health-Bulletin-2021.pdf> ) | 2021 | Addressed | Gender Blind |
| 7. Empirical and prophylactic use of antimicrobials- National guideline 2024 ( <https://slmicrobiology.lk/empirical-and-prophylactic-use-of-antimicrobials-national-guidelines-sri-lanka-2024/> ) | 2024 | Addressed | Gender Blind |
| 8. National Antimicrobial Stewardship Guideline for Healthcare Institutions ( <https://slmicrobiology.lk/wp-content/uploads/2024/10/National-Guideline-on-Antimicrobial-Stewardship-AMS-Programme-in-Healthcare-institutions-of-Sri-Lanka.pdf> ) | 2024 | Addressed | Gender Blind |
| 9. Implementation of AMR prescription chart in healthcare ( <https://slmicrobiology.lk/wp-content/uploads/2024/10/Prescription-chart.pdf> ) | 2024 | Addressed | Gender Blind |
| 10. implementation of Aware classification of AMR in Sri Lanka ( <https://slmicrobiology.lk/wp-content/uploads/2024/10/Implementation-of-Aware-classification-of-antimicrobials-in-healthcare-institutions-in-Sri-Lanka-General-Circular-No.-01-13-2024-the-list-of-AWaRe-antimicrobials.pdf> ) | 2024 | Addressed | Gender Blind |
