# Supplementary material for: Gender and equity within antimicrobial resistance: a scoping review of evidence and policy in Bangladesh and Sri Lanka
Source: BMC Public Health. 2026 Jun 4;26:2313. doi: 10.1186/s12889-026-27842-y (PMC13455460; doi:10.1186/s12889-026-27842-y)
Supplement: Supplementary file 8 [file 12889_2026_27842_MOESM6_ESM.docx]

**Additional File 6: Policy documents and grey articles in Bangladesh and Sri Lanka that not addressed AMR**

| **SL No** | **Nap/Policy/ Guideline/ Framework/report** | **AMR** | **Gender Responsive Assessment Scale** |
| --- | --- | --- | --- |
| 1 | National Healthcare Standards ( <http://qis.gov.bd/wp-content/uploads/2019/04/2017_02_26_1488073894_902rds_.pdf> ) | Not address AMR | Gender Blind |
| 2 | Bangladesh Environment policy ( <https://doe.portal.gov.bd/sites/default/files/files/doe.portal.gov.bd/page/5a9d6a31_d858_4001_b844_817a27d079f5/ENV%20POLICY%202018%20FINAL%20DRAFT.pdf> ) | Not addressed AMR | Gender Blind |
| 3 | Digital Health Strategy 2023-2027 of Bangladesh ( <https://dghs.portal.gov.bd/sites/default/files/files/dghs.portal.gov.bd/page/4124d18a_ab99_40e2_8fef_ff4052948739/2024-12-10-09-03-2c48d515dfda298717e1193ac4d41d49.pdf> ) | Not addressed AMR | Gender Blind |
| 4 | National Health Strategic Master Plan 2016-2025 ( <https://www.health.gov.lk/wp-content/uploads/2022/10/Health-Admin-HRH.pdf> ) | not addressed AMR | Gender Blind |
| 5 | National Medicines Regulatory Authority Act, No. 5 of 2015  ( <https://cdn.prod.website-files.com/666d0695ca3ba7fa496a5068/66cdaeb3175c53df7e905331_National%20medicines%20regulatory%20authority%20act%2C%20no.%205%20of%202015%20-%20Eng.pdf> ) | not addressed AMR | Gender Blind |
| 6 | The National Occupational Safety and Health Policy ( [https://www.health.gov.lk/wp-content/uploads/2022/10/25-National-Policy-on-Occupational-Shickafety-and-Health-Policy-2014-compressed.pdf](https://www.health.gov.lk/wp-content/uploads/2022/10/25-National-Policy-on-Occupational-Safety-and-Health-Policy-2014-compressed.pdf) ) | not addressed AMR | Gender Blind |
| 7 | National Policy on Health Information ( <https://www.health.gov.lk/wp-content/uploads/2022/10/21_Health-Information-Policy.pdf> ) | not addressed AMR | Gender Blind |
| 8 | National Health Policy 2016-2025 ( <https://www.health.gov.lk/wp-content/uploads/2022/08/2_National-Health-Policy-2016-2025_compressed.pdf> ) | Not addressed AMR | Gender Blind |
