# Supplementary material for: Gender and equity within antimicrobial resistance: a scoping review of evidence and policy in Bangladesh and Sri Lanka
Source: BMC Public Health. 2026 Jun 4;26:2313. doi: 10.1186/s12889-026-27842-y (PMC13455460; doi:10.1186/s12889-026-27842-y)
Supplement: Supplementary file 10 [file 12889_2026_27842_MOESM8_ESM.docx]

**Additional File 8:** WHO Gender Responsive Assessment Scale: criteria for assessing programmes and policies

| Level | Criteria | Level (Scoring Criteria) |
| --- | --- | --- |
| 1. Gender-unequal | - Perpetuates gender inequality by reinforcing unbalanced norms, roles and relations - Privileges men over women (or vice versa) - Often leads to one sex enjoying more rights or opportunities than the other | 1 |
| 2. Gender-blind | - Ignores gender norms, roles and relations - Very often reinforces gender-based discrimination - Ignores differences in opportunities and resource allocation for women and men - Often constructed based on the principle of being “fair” by treating everyone the same | 2 |
| 3. Gender-sensitive | - Considers gender norms, roles and relations - Does not address inequality generated by unequal norms, roles or relations - Indicates gender awareness, although often no remedial action is developed | 3 |
| 4. Gender-specific | - Considers gender norms, roles and relations for women and men and how they affect access to and control over resources - Considers women's and men's specific needs - Intentionally targets and benefits a specific group of women or men to achieve certain policy or programme goals or meet certain needs - Makes it easier for women and men to fulfil duties that are ascribed to them based on their gender roles | 4 |
| 5. Gender-transformative | - Considers gender norms, roles and relations for women and men and that these affect access to and control over resources - Considers women's and men's specific needs - Addresses the causes of gender-based health inequities - Includes ways to transform harmful gender norms, roles and relations - Objective is often to promote gender equality - Includes strategies to foster progressive changes in power relationships between women and men | 5 |

**Specific example below showing why a policy was rated “gender-blind":**

Bangladesh developed the National Action Plan (2017-2022) and National Action Plan (2020-2025) to address the alarming threat of antimicrobial resistance, prepared by the Disease Control Unit of the Directorate General of Health Services under the Ministry of Health and Family Welfare (MoHFW). This NAPs emphasizes on the rational use of antibiotics, establishing the standard treatment guidelines, antibiotic stewardship programs, establishment of a comprehensive surveillance system including human, animal and environment, good manufacturing practice (GMP), development of a microbiology laboratory, and promoting research on AMR. However, there is no mention of gender or sex in the paper. Gender-specific interventions, gender-disaggregated data, and structural gender disparities (e.g., gender-based occupational exposures, social norms impacting antibiotic usage, or differential access to health care by gender) are not included. Given these reason, we scored Bangladesh NAP as “gender-blind.”

Source: World Health Organization, 2010

**Reference:**

Gender mainstreaming for health managers: a practical approach. Participant's notes. Geneva: World Health Organization; 2011. (<https://apps.who.int/iris/bitstream/handle/10665/44516/9789241501064_eng.pdf?sequence=2>, accessed 06 October 2025)
