# Supplementary material for: Gender and equity within antimicrobial resistance: a scoping review of evidence and policy in Bangladesh and Sri Lanka
Source: BMC Public Health. 2026 Jun 4;26:2313. doi: 10.1186/s12889-026-27842-y (PMC13455460; doi:10.1186/s12889-026-27842-y)
Supplement: Supplementary file 11 [file 12889_2026_27842_MOESM9_ESM.docx]

| **Citations** | **Characteristics of included articles** | **Summary of findings** | | | | |
| --- | --- | --- | --- | --- | --- | --- |
|  |  | **Susceptibility to infection** | **Exposure to infection** | **Antimicrobial Use** | **Impacts (Stigma/ norms)** | **Recommendation** |
|  | - Study design - Study site - Sample | - Clinical susceptibility - Anatomical susceptibility - Environmental - Malnutrition | - Occupational exposure - Exposure in homes and communities | - Access/ choice to healthcare and resources   - Physical access barriers   - Financial barriers   - Decision-making autonomy - Distribution of labor, practices, and role (Household level) - Decision-making power and Autonomy - Prescription needed/ pattern - Administration (medicine dose) - Self-medication - Education and knowledge related to AMR / source of information - Attitudes towards illness and medicine |  |  |

**Purpose of Data extraction form:** The purpose of data extraction form is to chart, organize and extract all the relevant information from peer-reviewed articles which are included in the scoping review. The form ensures consistency of data extracting.

**How it aligns with review objectives and how intersectional factors were operationalized:**

The data extraction form directly aligned with the scoping review objectives and review questions.

Here, study characteristics help to identify the gaps and evidence available regarding various contextual and methodologically settings. It also give us the valuable insights like which study analyze the data base on gender segregation.

From the extracted data, we identified the key findings of the review that integrate the evidence from peer-reviewed articles and illustrate how gender, equity, and other intersectional factors such as occupation, financial condition, educational status, age, and anatomical structure influence AMR. Here, the significant and relevant findings are organized into six key themes: i) Gender-based knowledge, attitude, and practice regarding AMR ii) Gender disparities in non-recommended antibiotic use and sources iii) Influence of gender roles and norms in AMR iv) Gender-based vulnerability to infection and exposure to AMR v) Role of Healthcare Providers and Prescription Patterns in AMR. To strengthen transparency and thematic coherence, the six emergent themes were explicitly mapped to the five research questions presented in Table 1. Themes 1 to 4 address gender roles, norms, and equity as they influence knowledge, attitudes, practices, and intersectional disparities related to AMR which aligns with research question 1 and 2. Themes 5 reflect the gendered perspectives of both formal and informal healthcare providers and their influence on treatment-seeking and antibiotic use (research question 3 and 4). Finally, the policy document analysis corresponds to research question 5 by examining the extent to which national AMR policies in Bangladesh and Sri Lanka incorporate gender and equity considerations which further explain in result sections and additional file 4 & 5.
